# Digital multiplexed mRNA analysis of functionally important genes in single human oocytes and correlation of changes in transcript levels with oocyte protein expression^☆^

**DOI:** 10.1016/j.fertnstert.2013.11.125

**Published:** 2014-03

**Authors:** Solon Riris, Philippa Webster, Hayden Homer

**Affiliations:** a Reproductive Medicine Unit, Elizabeth Garrett Anderson Wing, University College London Hospitals, London, United Kingdom; b NanoString Technologies, Seattle, Washington; c Institute for Women’s Health, University College London, London, United Kingdom; d School of Women's & Children's Health, University of New South Wales, Randwick, New South Wales, Australia

**Keywords:** NanoString, human oocytes, gene expression, expression profiling, oocyte quality

## Abstract

**Objective:**

To investigate functionally important transcripts in single human oocytes with the use of NanoString technology and determine whether observed differences are biologically meaningful.

**Design:**

Analysis of human oocytes with the use of NanoString and immunoblotting.

**Setting:**

University-affiliated reproductive medicine unit.

**Patients:**

Women undergoing in vitro fertilization.

**Intervention:**

Human oocytes were analyzed with the use of NanoString or immunoblotting.

**Main Outcome Measures:**

The abundance of transcripts for ten functionally important genes—*AURKA, AURKC, BUB1, BUB1B* (encoding BubR1), *CDK1, CHEK1, FYN, MOS, MAP2K1,* and *WEE2—*and six functionally dispensable genes were analyzed with the use of NanoString. BubR1 protein levels in oocytes from younger and older women were compared with the use of immunoblotting.

**Result(s):**

All ten functional genes but none of the six dispensable genes were detectable with the use of NanoString in single oocytes. There was 3- to 5-fold variation in *BUB1*, *BUB1B*, and *CDK1* transcript abundance among individual oocytes from a single patient. Transcripts for these three genes—all players within the spindle assembly checkpoint surveillance mechanism for preventing aneuploidy—were reduced in the same oocyte from an older patient. Mean *BUB1B* transcripts were reduced by 1.5-fold with aging and associated with marked reductions in BubR1 protein levels.

**Conclusion(s):**

The abundance of functionally important transcripts exhibit marked oocyte-to-oocyte heterogeneity to a degree that is sufficient to affect protein expression. Observed variations in transcript abundance are therefore likely to be biologically meaningful, especially if multiple genes within the same pathway are simultaneously affected.

**Discuss:** You can discuss this article with its authors and with other ASRM members at **http://fertstertforum.com/ririss-oocyte-quality-gene-expression/**

Oocytes provide the overwhelming majority of cytoplasmic building blocks for early embryogenesis. Consequently, a major determinant of pregnancy success is oocyte quality. Yet surprisingly little is known about its important molecular determinants. In large part, this stems from the extreme paucity of human oocytes available for research, making approaches capable of single-oocyte analysis especially appealing.

Many studies have now examined gene expression at the mRNA level in human oocytes (1–5). Moreover, by incorporating an amplification stage, analyses have been extended to single human oocytes (6, 7) and even single polar bodies (8). Notably, however, the functions of the overwhelming majority of genes reported in those studies have not previously been examined specifically in oocytes but are instead inferred from their roles in mitotic (somatic) cells. Furthermore, it is not known whether the differences observed in mRNA abundance between distinct groups of patients, such as older versus younger, have any meaningful impact on protein abundance, the downstream mediator of gene function. Nor has it been explored how deregulated transcripts within the same oocyte might relate to one another, for example, by acting within a common regulatory pathway.

The NanoString nCounter system is a new technology for digitally estimating mRNA abundance with the use of unique color-coded probes (9). NanoString is more sensitive than microarrays and of similar sensitivity to qPCR (9) and is now used to validate microarrays (10) and next-generation sequencing technology (11). NanoString has not previously been applied to human oocytes.

Herein, we used NanoString technology to analyze functionally important genes in single human oocytes. We identify striking “in-patient” oocyte-to-oocyte heterogeneity in key genes. Moreover, the extent of variation observed for one such gene, *BUB1B*, is associated with marked reductions in total oocyte levels of the encoded BubR1 protein. Interestingly, transcripts for *BUB1* and *CDK1* were also found to be reduced along with *BUB1B* within the same oocyte. Given that all three genes are pivotal players within the spindle assembly checkpoint (SAC) signaling pathway for ensuring accurate chromosome segregation, the potential disruptive consequences could be exponentially greater.

## Materials and methods

### Source of Human Oocytes and Ethical Approval

Human oocytes were obtained from women undergoing in vitro fertilization (IVF; with or without intracytoplasmic sperm injection) at the IVF Unit at University College London Hospitals. Written patient consent was obtained after ethical approval was received from the National Research Ethics Service Committee London (REC reference 11/LO/1360). All women were <40 years old, had a body mass index (BMI) <30 kg/m^2^, were nonsmokers, and met eligibility criteria for National Health Service–funded treatment. Procedures used for IVF have been described previously (12).

Oocytes used for nCounter analyses were obtained from 14 women (age-range 30–39 years). Oocytes for immunoblotting were obtained from 12 women, five who were <32 years (“young”) and seven who were >37 years (“older”).

### Human Oocyte Samples

Two nCounter assays involving a total of 39 oocytes were undertaken. Each assay has the capacity for simultaneously analyzing 12 samples.

One assay analyzed triplicate samples of five and three pooled human oocytes at the metaphase II (MII)–arrested stage (5- and 3-oocyte samples), quadruplicate samples of single MII-stage oocytes (single-oocyte samples) and duplicate samples of single oocytes at the germinal vesicle (or GV) stage (Supplemental Table 1; Supplemental Tables 1-3 are available online at www.fertstert.org). Two of the 5-oocyte samples were derived from one patient each, whereas the third sample was composed of oocytes pooled from two patients. The 3-oocyte samples were derived from one patient each. Two of the single-oocyte samples were from the same patient, whereas the other two were from two different patients. Both GV-stage oocytes were from the same patient.

The other NanoString assay included triplicate single-oocyte samples from each of two patients, triplicate samples in which no oocytes were added but were of otherwise identical volume and chemical composition (termed “empty” samples), and three oocytes from a single patient that were lysed together in one larger-volume sample before being divided into three equal volumes (termed “one-third” samples) (Supplemental Table 2; Supplemental Tables 1–3 are available online at www.fertstert.org). These one-third samples function as an indicator of any potential assay to assay variability, which is not normalized away by the positive control normalization (see later); differences in results likely reflect sample pipetting inaccuracies.

Oocytes used for immunoblotting were all at the MII stage.

GV-stage oocytes were obtained ∼40 h after hCG administration. MII-stage oocytes comprised failed-to-fertilize oocytes, the determination of which was made 18–20 h after insemination. Cumulus-free oocytes were washed free of culture medium with the use of 1% polyvinyl pyrolidone (Sigma) and lysed either in RLT buffer (Qiagen) to make a final sample volume of 5 μL for NanoString analyses or in LDS sample buffer (Nupage; Invitrogen) in pools of ten for immunoblotting. Lysates were snap-frozen at −80°C.

### NanoString nCounter Analyses

The nCounter assay (NanoString Technologies) involves hybridizing target sequences in the sample by complementary base pairing to a pair of gene-specific probes. Each probe pair is composed of a reporter probe (bearing a unique color barcode derived from a specific configuration of four possible colours at six positions) and a biotinylated capture probe so that hybridization results in the production of tripartite probe-target complexes in solution. We used an off-the-shelf Codeset, the nCounter GX Human Kinase Panel (NanoString Technologies), containing probe pairs directed against 528 human kinase-encoding genes and 8 reference genes (identified by bold italics in Supplemental Tables 1 and 2). Hybridizations were carried out according to the NanoString Gene Expression Assay manual. Each 5-μL oocyte sample in RLT buffer was mixed directly with 10 μL nCounter reporter probes, 5 μL nCounter capture probes, and 10 μL hybridization buffer for a total reaction volume of 30 μL. The hybridizations were incubated at 65°C for 16–20 h.

Following hybridization, a custom liquid-handling robot, the nCounter Prep Station, was used to remove excess probes by affinity purification. The tripartite complexes were then bound via their biotinylated capture probes to the streptavidin-coated surface of a sample cartridge, electrophoresed to elongate and align the complexes, and then immobilized in preparation for analysis. The end-product at this stage is a series of immobilized mRNA transcripts derived from the sample, each tagged with a specific color barcode corresponding to a particular gene transcript. Subsequently, the cartridges were placed in the nCounter Digital Analyzer for fully automated imaging and data collection. The expression level of a gene was determined by counting the number of times its specific barcode was detected.

Positive control normalized nCounter data are normalized to the average of the counts of a titrated series of six synthetic RNA transcripts that are spiked into every hybridization reaction. Normalization to these internal positive control samples, which are provided with the assay reagents, account for slight differences in assay efficiency (hybridization, purification, binding, etc.). Concentrations of the control transcripts range from 0.125 fM to 128 fM. In a typical nCounter assay, a second normalization to the expression of appropriate reference (or “housekeeping”) genes to control for sample input can also be performed. We focused our analyses on positive control normalized data because we added defined numbers of oocytes directly to the assay without RNA purification, thereby reducing the likelihood of any meaningful variation in sample input. Positive control normalized data from both assays are presented for all genes in the GX Human Kinase Panel in Supplemental Tables 1 and 2. As described in greater detail later, this paper focused on a subset of ten genes extracted from the Kinase Panel that were previously shown to be functionally important specifically within the context of oocytes and seven functionally dispensable genes.

### Criteria for Detection with the Use of NanoString

NanoString incorporates eight spiked-in negative control probe sets that have no corresponding targets within the sample and give a readout of background noise in the system. By convention, a gene is considered to be detected if its absolute count is higher than 2 standard deviations above the mean count of the spiked-in negative control samples (10). The detection threshold was calculated to be 23 in both of the assays undertaken in the present study. We further considered that for detection the gene should be above this threshold level in at least one sample from every patient.

### Immunoblotting

Immunoblotting was performed as detailed previously (13–16). In short, proteins were resolved on 4%–12% Bis-Tris gels (Nupage; Invitrogen) before being transferred to polyvinylidene difluoride membranes (Millipore). Membranes were blocked for 1 h at room temperature (RT) in 3% bovine serum albumin (BSA; Sigma) before probing with a sheep polyclonal anti-BubR1 primary antibody (a kind gift from Professor Stephen Taylor, University of Manchester) overnight at 4°C, followed by an horseradish peroxidase–conjugated antisheep secondary antibody (Sigma) for 1 h at RT. Actin (Millipore) was used as a loading control. Detection was performed with the use of ECL Plus (GE Healthcare) ,and images were captured with the use of a Chemidoc XRS Imaging System (Bio-Rad).

### Determination of Gene Functionality

We reasoned that those genes shown to be functionally important in oocytes would be most informative regarding oocyte quality.

*CDK1* (for cyclin-dependent kinase 1) (17) and *MOS* (a MAP kinase kinase kinase [or MAP3K] acting in a pathway with *MAP2K1*) (18) are firmly established as universal and well conserved regulators of mammalian oocyte maturation and were obvious genes of interest. We identified seven additional genes from the Human Kinase Panel that have been found to be important in mouse oocytes with the use of gene-targeting strategies (Supplemental Table 3). These seven genes included *Aurora kinase A* (*AURKA*) (19, 20), *Aurora kinase C* (*AURKC*) (21, 22), *BUB1* (23–25), *BUB1B* (15, 26, 27), *CHEK1* (28, 29), *FYN* (30, 31), and *WEE2* (or *WEE1B*) (32, 33). Importantly, all of these genes are expressed in human oocytes (1, 3, 5). Moreover, both *AURKC* (34) and *FYN* (35) have also recently been studied at the protein level in human oocytes.

We also identified six genes from the Human Kinase Panel which, in contrast to the above functionally important genes, have been shown to be functionally dispensable in oocytes. Unlike the oocyte-specific *WEE2* (32), there is a distinct mitotic form known as *WEE1* that is important in somatic cells but dispensable in oocytes (33). In stark contrast to CDK1, other CDKs, such as CDK2, CDK3, CDK4, and CDK6, are dispensable for oocyte maturation (36). Unlike MOS, another MAP3K, RAF1, does not play an important role in the MAPK cascade in oocytes (37).

In summary therefore, we focused on ten functionally important genes—*AURKA*, *AURKC*, *BUB1*, *BUB1B*, *CDK1*, *CHEK1*, *FYN*, *MAP2K1*, *MOS*, and *WEE2*—and six functionally dispensable genes—*CDK2*, *CDK3*, *CDK4*, *CDK6*, *RAF1*, and *WEE1*.

### Statistical Analyses

Statistical analyses were performed with the use of Graphpad Instat software. A *P* value of <.05 with the use of the Student *t* test was considered to be statistically significant.

## Results

### Detection of Multiple Transcripts in Single Human Oocytes with the Use of NanoString

We found that transcripts for all ten functionally important genes were detectable (Table 1). *AURKA*, *BUB1B*, *CHEK1*, *FYN*, *MAP2K1*, *MOS*, and *WEE2* were detected in all ten MII-stage single-oocyte samples, *AURKC* and *CDK1* were detectable in nine of them, and *BUB1* in eight (Table 1). Overall, therefore, 96 out of 100 reads exceeded the detection threshold (Table 1).

**Figure 1:**
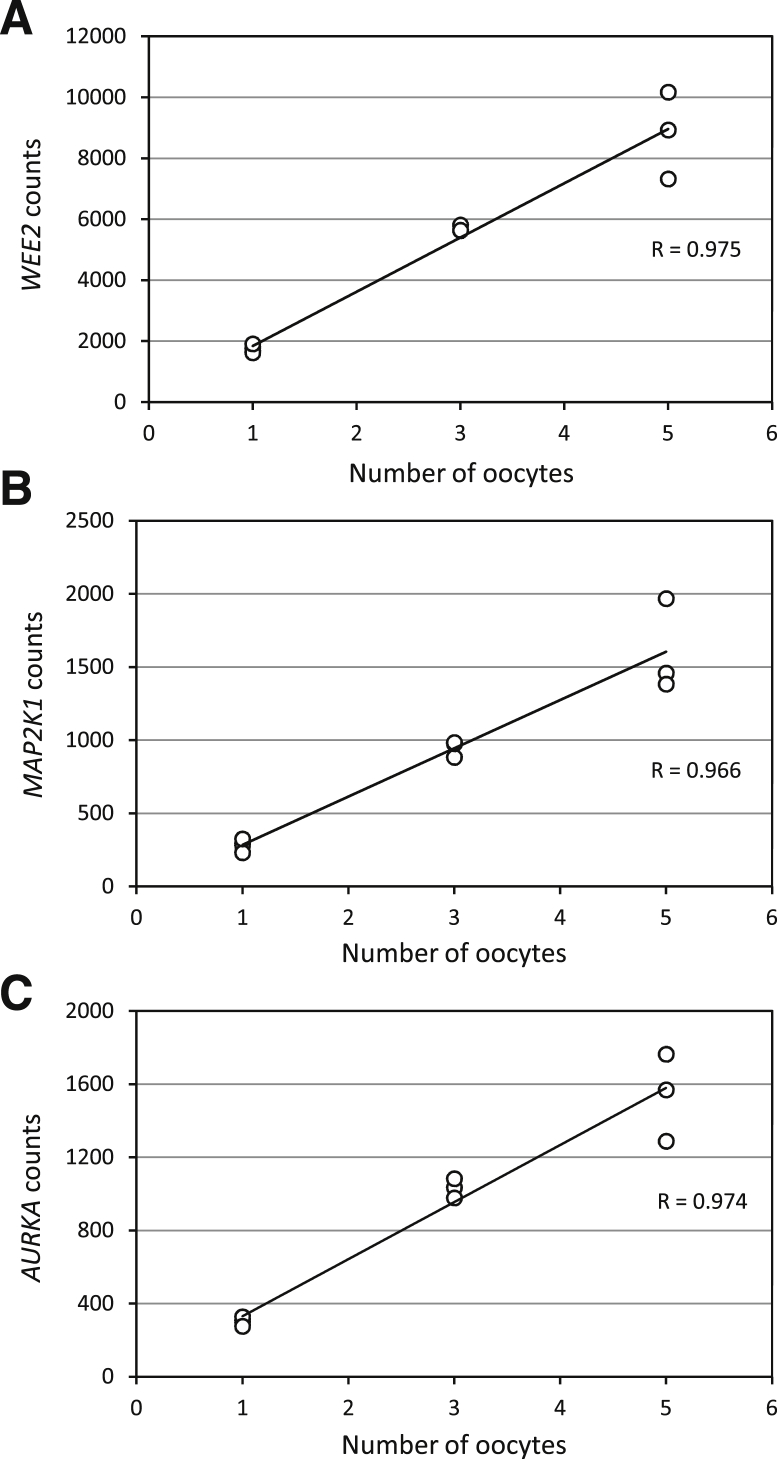
Correlation between counts for (A) *WEE2*, (B) *MAP2K1*, and (C) *AURKA* and numbers of oocytes. *R* was used to calculate the Pearson correlation coefficient.

We compared counts for the single-oocyte samples with those for the 5-oocyte and 3-oocyte samples and found that counts increased in relation to sample input for the functional genes (Supplemental Table 1). Indeed, further analysis of *WEE2*, *MAP2K1*, and *AURKA*, the three kinases with the highest counts, revealed a very high degree of correlation (*R* > 0.96) between counts and sample input (Fig. 1).

In marked contrast to the ten functional genes, none of the six dispensable genes—*CDK2, CDK3, CDK4, CDK6, RAF1,* and *WEE1*—crossed the detection threshold in any of the single-oocyte samples (Table 1). Furthermore, counts for these six genes were similar to those found in empty samples (Table 1) and remained undetectable in the 3- and 5-oocyte samples (Supplemental Table 1).

Transcript abundance declines between the GV and MII stages in both mouse (38) and human (3, 39) oocytes. Entirely consistent with this, we found that transcript abundance for eight of the ten functional genes was significantly higher at the GV stage than at the MII stage, whereas all functionally dispensable genes remained undetectable at all stages (Table 1).

### Variation in Transcript Abundance among Individual Oocytes and Genes

We analyzed the counts of 3 oocytes we obtained from each of two patients (patients 4 and 5; Table 1). Counts for the 10-gene panel were on average higher in oocytes from patient 4 than in those from patient 5 (Table 2). Along with overall higher counts for oocytes from patient 4, there was also more oocyte-to-oocyte consistency, with no transcript exhibiting >1.5-fold difference in abundance from one oocyte to the next (range 1.1–1.4-fold; Table 2). In stark contrast, among oocytes from patient 5, counts for three genes (*BUB1*, *BUB1B*, and *CDK1*) exhibited 3- to 5-fold differences, and *MOS* varied by more than 2-fold (Table 2).

The marked variation in transcript abundance for patient 5 but not for patient 4 pointed to patient-specific variation rather than inherent test instability. To examine this further, we investigated “one-third” samples (three oocytes from a third patient, patient 6, lysed together and divided into three equal volumes), which were run on the same assay as oocytes from patients 4 and 5 (Supplemental Table 2). Mean transcript abundance for more than one-half of the genes in oocytes from patient 6 were higher than those from either patient 4 or patient 5, again reaffirming interpatient differences (Table 2). Notably, however, none of the genes in the one-third samples showed >1.5-fold difference in counts (Table 2), confirming that patient 5’s oocyte-to-oocyte variability did not reflect inherent assay properties.

We found wide variation in transcript abundance from one gene to the next within individual oocytes. Thus, at the high-abundance end of the spectrum was *WEE2*, with counts ranging from 1,474 to 2,124, roughly 6-fold higher than the next most abundant kinase. In contrast, at the low end of the range were genes such as *BUB1* with counts from 13 to 52, roughly 50- to 100-fold lower than *WEE2*.

### Age-Related Changes in BubR1 Protein Levels

**Figure 2:**
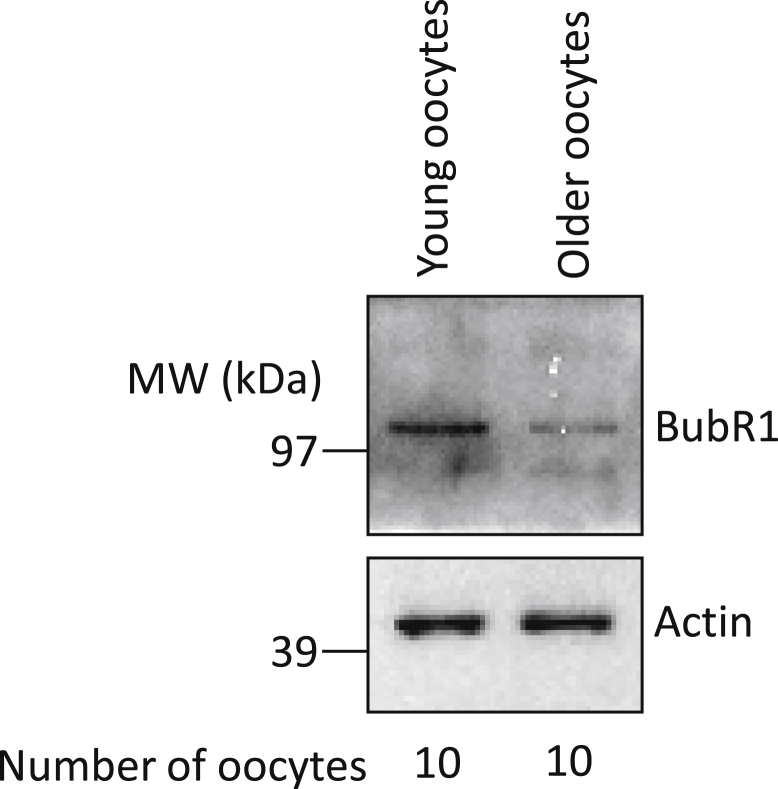
Immunoblot of BubR1 and Actin in lysates of oocytes from young (<32 years) and older (>37 years) women.

We observed a 1.5-fold reduction in mean *BUB1B* transcript levels between a 39-year-old (patient 5) and a 31 year-old (patient 4; Table 2). This is similar to the 1.42-fold reduction observed previously between <32-year-olds and >40-year-olds with the use of microarrays (40). We investigated whether these changes in transcript abundance corresponded with discernible changes in cognate protein levels by immunoblotting ten oocytes from women ≤32 years old alongside ten oocytes from women >37 years old. We found that the BubR1 signal in the older age group was markedly reduced compared with the younger age group, whereas the signal for the actin loading control was indistinguishable (Fig. 2). Thus, a ∼1.5-fold transcript decline is associated with marked reduction in protein expression.

## Discussion

We used NanoString to investigate, for the first time, human oocytes and found that we could detect a panel of ten functionally important genes in a single oocyte. This capability was validated by data showing that counts were inextricably linked with gene functionality (dispensable genes were uniformly undetectable), that counts correlated very strongly with oocyte numbers, and that counts demonstrated predicted changes regarding maturation stage. We also note that mean *AURKA*, *BUB1B*, and *CHEK1* counts were, respectively, 1.3-, 1.5-, and 1.4-fold lower for patient 5 (39 years old) than for patient 4 (31 years old; Table 2) and that previous microarray data showed 1.72-, 1.42-, and 1.35-fold decreases, respectively, in these same transcripts between pooled oocytes from young women (aged <32 years) and older women (aged >40 years) (40). Therefore, although age-related changes in transcript abundance was not a primary aim of our study, it is nevertheless noteworthy that age-related changes in counts we observed here are very similar in magnitude to those reported previously for microarrays (40), providing further validation for NanoString.

Strikingly, there was marked variation in transcript abundance for key genes among oocytes from a given individual. This new finding is distinct from the variations in global gene expression profiles recently reported for individual oocytes from different patients (7). Also interesting was the wide variation in relative transcript abundance within a single oocyte. Genes such as *BUB1* and *AURKC* are at one extreme with counts per oocyte generally less than 50 whereas at the other extreme are genes such as *WEE2* with counts well above 1,000.

We wanted to determine whether the observed changes in transcript abundance might be significant enough to affect gene function. We elected to examine *BUB1B* because previous microarray data for both humans (40) and mice (41), along with the present NanoString results, indicated that oocyte *BUB1B* transcripts consistently decline with age by around 1.5-fold. We examined whether this degree of change in *BUB1B* affected BubR1 protein levels by immunoblotting oocytes from younger and older patients, and we found marked reductions in BubR1 levels in older oocytes (Fig. 2). This is highly significant, because we and others previously found that even modest reductions in BubR1 levels can severely compromise BubR1 function and affect oocyte maturation (15, 27). Interestingly, chromosome misalignment (42, 43) and missegregation (44) are prominent features of both aged human oocytes and BubR1-depleted mouse oocytes (15, 26, 27), suggesting that compromised BubR1 function could be an important contributor to poor oocyte quality. Given that ∼1.5-fold transcript reduction affected protein expression, it is very possible that the 3- to 5-fold oocyte-to-oocyte variation in transcript abundance that we observed could have significant implications for the function of a wider range of genes. The defect incurred could be even greater still, because *BUB1B*, *BUB1*, and *CDK1* transcripts all exhibited reductions within the same oocyte. Because all three are key components of the SAC pathway, which is critical for preventing aneuploidy (15, 23–27), the cumulative effect could severely disrupt chromosome segregation fidelity in that oocyte.

The present report focused specifically on genes that have been shown to be functional in the mouse oocyte model (Supplemental Table 3). Although we acknowledge that the functionality of most of these genes has not been corroborated directly in human oocytes, it must also be acknowledged that this is impossible to achieve with the same level of rigor as can be achieved with mouse oocytes owing to the extreme dearth of biological material. Significantly however, all of the ten genes studied have been shown to be expressed in human oocytes, and for some genes, such as *AURKC* and *FYN*, more direct parallels have been drawn between mouse and human oocytes (34, 35). Added to this, there is robust evidence that the cyclic adenosine monophosphate–based pathway modulating CDK1 activity through WEE1 kinases (45) is conserved between mouse and human oocytes (46).

Other emerging data further underscore the relevance of the mouse model to understanding human oocyte regulation. Thus, like women, female mice exhibit age-related fertility decline linked to oocyte-derived aneuploidy and compromised integrity of the molecular chromosomal “glue” known as cohesin (47, 48). Other examples common to mouse and human oocytes include the deacetylation of chromatin-associated histones during maturation which is also vulnerable to aging (49, 50) and the age-related accumulation of oocyte DNA damage (51). Mouse and human oocytes also show striking parallels regarding the profile of transcripts that become deregulated with age. In both sets of oocytes, *BUB1B* and *CHEK1* transcripts decline by similar magnitudes (40, 41). Other examples of overlap include DNA repair genes, such as *BRCA1* (51), as well as the p53 family member *TAp73* (52), which interestingly, has been shown to be an upstream regulator of BubR1 in oocytes critical for female fertility (53, 54).

Ethical considerations restricted us to using MII-stage oocytes that had failed to fertilize. Although such oocytes might not be considered to be representative of oocytes that support fertilization, it does not detract from our findings that NanoString can profile multiple transcripts in a single human oocyte or that observed variations in transcript abundance are likely to be biologically meaningful. It is important to note that the oocyte-to-oocyte heterogeneity we identified here pertains to a very uniform cohort of oocytes: All failed to fertilize, all were derived from a single patient and were therefore “internally” controlled regarding their genetic and infertility background, and all were subjected to identical culture conditions and sample preparation. This heterogeneity does not reflect inherent test instability either, because it was not evident for all patients and, importantly, it was not observed in the one-third samples. Based on these considerations, one might predict even greater differences if comparisons were to be made between the failed-to-fertilize cohort and oocytes that undergo fertilization. Such differences, if indeed more marked, could be clinically helpful for selecting the most developmentally competent oocytes.

## Figures and Tables

**Table 1:** nCounter counts for single-oocyte samples.

	1^a^		2	3	4			5			Empty	Empty	Empty	GV	GV	*P*^b^
	MII	MII	MII	MII	MII	MII	MII	MII	MII	MII						
*AURKA*	295	309	328	276	328	378	391	254	357	240	1	2	0	475	484	.0004
*AURKC*	32	24	33	65	44	42	43	23^c^	36	32	1	2	1	35	57	.6416
*BUB1*	37	34	28	31	32	30	29	13^c^	52	18^c^	0	2	1	69	64	.0006
*BUB1B*	133	132	101	118	156	140	130	86	153	48	2	1	0	217	259	.0025
*CDK1*	58	80	65	56	92	64	83	46	111	23^c^	0	0	2	168	160	.0003
*CHEK1*	83	107	102	107	86	100	96	53	87	61	0	0	0	117	142	.0633
*FYN*	75	93	72	81	100	72	81	57	97	52	0	0	2	126	190	.0193
*MAP2K1*	295	282	231	325	243	291	268	188	275	214	1	1	0	460	449	.0044
*MOS*	46	69	71	105	81	93	82	36	63	28	1	1	4	171	220	.0066
*WEE2*	1,758	1,639	1,620	1,911	1,975	1,963	2,124	1,474	1,821	1,372	2	0	0	2,645	2,662	.0008
*CDK2*	3	2	2	1	2	1	0	2	2	0	0	0	0	4	4	
*CDK3*	0	3	2	2	0	0	0	0	1	0	0	0	0	1	2	
*CDK4*	4	3	3	5	2	3	3	2	2	2	1	2	0	6	6	
*CDK6*	3	3	0	1	1	2	0	1	0	1	2	2	1	1	3	
*RAF1*	1	0	1	1	0	0	1	1	0	0	0	0	0	2	1	
*WEE1*	1	0	1	1	1	0	0	0	1	0	0	1	1	2	8	

a Numbers above columns identify different patients.

b *P* values refer to statistical comparisons between counts in GV-stage (GV) oocytes and counts in MII-stage (MII) oocytes.

c Reads that do not exceed the detection threshold of 23.

**Table 2:** Inter- and intrapatient variation in nCounter counts.

	Single-oocyte samples				Ratio of means	One-third samples	
	Patient 4 (31 y)		Patient 5 (39 y)			Patient 6	
	Mean^a^	Fold change^b^	Mean^a^	Fold change^b^		Mean^a^	Fold change^b^
*AURKA*	366	1.2	284	1.5	1.3	450	1.1
*AURKC*	43	1.1	30	1.6	1.4	73	1.5
*BUB1*	30	1.1	28	4.0	1.1	42	1.2
*BUB1B*	142	1.2	96	3.2	1.5	177	1.5
*CDK1*	79	1.4	60	4.9	1.3	118	1.3
*CHEK1*	94	1.2	67	1.7	1.4	105	1.1
*FYN*	84	1.4	69	1.9	1.2	130	1.3
*MAP2K1*	267	1.2	226	1.5	1.2	348	1.1
*MOS*	86	1.1	42	2.3	2.0	136	1.5
*WEE2*	2,020	1.1	1,556	1.3	1.3	2,188	1.1

a Mean of counts from the three samples analyzed per patient (see Table 1).

b Fold change between maximum and minimum counts obtained for each patient (see Table 1).
