# Supplementary material for: Digital multiplexed mRNA analysis of functionally important genes in single human oocytes and correlation of changes in transcript levels with oocyte protein expression
Source: Fertil Steril. 2014 Mar;101(3):857–64. doi: 10.1016/j.fertnstert.2013.11.125 (PMC3969224; doi:10.1016/j.fertnstert.2013.11.125)
Supplement: Supplemental Table 1 [file mmc1.pdf]

Positive Control Normalised Counts

Oocyte Nos.	5-oocytes			3-oocytes			Single-oocytes					
Stage	MII	MII	MII	MII	MII	MII	MII	MII	MII	MII	GV	GV
Target												
POS_A(128)	33744	33772	33785	33520	33710	33751	33614	33421	33502	33481	33492	33680
POS_B(32)	5718	5883	5774	5946	5757	5834	5930	6002	5963	5990	6008	5818
POS_C(8)	2202	2128	2153	2222	2283	2175	2113	2258	2195	2242	2236	2226
POS_D(2)	513	453	492	524	477	449	545	497	537	493	483	484
POS_E(0.5)	178	128	148	154	150	154	165	184	165	155	148	159
POS_F(0.125)	35	27	39	24	13	27	23	29	28	28	23	24
NEG_A(0)	0	0	0	0	1	0	3	1	0	1	0	0
NEG_B(0)	3	5	3	2	4	1	4	2	2	3	3	2
NEG_C(0)	4	4	2	2	5	6	3	1	3	5	4	0
NEG_D(0)	23	34	27	31	24	22	45	23	28	22	32	21
NEG_E(0)	2	3	0	0	2	2	1	0	1	0	0	1
NEG_F(0)	0	3	4	4	2	1	2	2	3	0	2	2
NEG_G(0)	1	0	1	1	0	0	0	0	1	1	0	0
NEG_H(0)	2	4	3	1	4	2	1	2	1	1	2	2
AAK1	31	63	25	25	28	31	12	4	11	22	28	17
AATK	1	2	0	0	1	0	1	2	1	1	2	2
ABL1	122	187	119	91	85	90	28	37	37	34	63	53
ABL2	68	79	80	69	44	49	7	12	11	19	28	26
ACVR1	35	56	59	18	25	27	6	7	11	5	15	21
ACVR1B	339	436	334	211	213	187	65	69	84	55	85	125
ACVR1C	2	4	2	0	0	3	2	1	0	0	2	0
ACVR2A	68	82	70	55	60	69	7	4	19	11	26	43
ACVR2B	118	183	149	97	108	119	19	18	38	42	40	47
ACVRL1	8	8	4	4	1	5	3	3	5	3	3	2
ADCK1	3	4	2	4	1	2	3	2	2	1	0	3

ADCK2	78	101	83	53	55	44	13	7	26	16	35	63
ADCK3	9	22	14	8	7	11	3	5	1	2	5	10
ADCK4	4	2	0	0	4	2	1	1	3	1	2	1
ADCK5	2	3	2	1	2	3	1	0	2	0	0	5
ADRBK1	190	199	208	104	154	140	24	50	38	31	64	62
ADRBK2	34	51	42	33	40	24	3	9	7	18	9	20
AKT1	213	304	189	149	166	137	33	51	52	30	53	60
AKT2	284	420	298	232	208	216	55	63	58	64	131	151
AKT3	305	388	354	255	262	207	74	53	75	81	114	116
ALK	0	1	2	0	1	0	0	1	1	0	3	1
ALPK1	2	3	1	3	1	2	0	1	3	1	0	1
ALPK2	1	2	3	5	0	0	0	0	1	0	0	1
ALPK3	2	3	1	1	0	3	0	0	1	1	1	3
AMHR2	3	1	4	2	2	0	2	1	0	3	0	0
ANKK1	4	2	0	0	4	0	3	1	0	0	1	1
ARAF	32	51	22	16	23	24	6	6	10	5	14	17
ATM	4	5	4	1	4	3	0	1	0	1	0	2
ATR	34	57	26	23	35	26	12	6	5	9	14	13
AURKA	1288	1764	1569	1034	1083	978	295	309	328	276	475	484
AURKB	120	160	157	127	89	111	31	23	20	42	52	65
AURKC	363	169	224	112	117	204	32	24	33	65	35	57
AXL	5	6	1	1	0	6	0	0	3	2	3	2
BCKDK	25	36	32	16	10	18	4	3	6	7	9	18
BCR	125	237	168	88	101	87	43	43	31	33	51	63
BLK	25	29	20	18	18	8	3	3	8	1	6	14
BMP2K	76	109	108	71	96	92	16	15	20	33	55	61
BMPR1A	287	455	339	216	237	239	74	81	77	61	115	134
BMPR1B	31	54	46	43	28	15	17	9	8	7	28	32
BMPR2	81	125	90	46	46	58	21	18	12	22	26	40
BMX	3	0	1	0	0	3	0	1	3	2	2	4
BRAF	35	38	42	24	18	19	14	6	8	10	17	17
BRD2	240	301	232	161	145	170	42	39	49	57	69	59
BRD3	65	86	58	45	63	58	12	16	17	24	27	24

BRD4	373	519	371	271	296	304	87	84	86	74	112	121
BRDT	61	105	54	27	37	37	11	5	11	10	27	42
BRSK1	2	2	3	1	2	3	3	2	3	3	0	3
BRSK2	0	1	0	1	1	1	1	0	0	0	0	0
BTK	1	0	1	0	0	0	0	1	1	0	0	0
BUB1	135	221	145	99	106	106	37	34	28	31	69	64
BUB1B	572	801	618	445	383	392	133	132	101	118	217	259
C21orf7	166	266	174	101	114	120	41	32	14	44	117	132
C9orf96	0	3	2	1	0	2	0	0	0	1	1	2
CAMK1	0	3	2	2	1	2	2	1	2	2	2	2
CAMK1D	43	46	33	30	31	31	6	3	9	18	15	11
CAMK1G	0	1	0	0	0	2	2	0	0	1	1	1
CAMK2A	10	28	11	8	23	11	6	5	1	4	13	11
CAMK2B	1	1	0	3	0	0	1	0	2	0	0	4
CAMK2D	4	4	7	4	1	2	3	4	2	1	0	2
CAMK2G	195	241	163	113	131	101	34	43	28	37	65	83
CAMK4	30	29	26	12	28	19	8	7	9	8	11	18
CAMKK1	10	12	10	5	7	7	3	1	3	1	3	6
CAMKK2	9	13	12	8	4	7	3	3	3	1	6	8
CAMKV	2	2	1	1	1	1	1	0	0	0	1	1
CASK	3	2	1	1	1	0	0	1	1	1	0	0
CCL2	8	4	3	5	1	6	6	5	7	4	6	5
CDC42BPA	57	101	77	45	42	71	8	9	16	12	32	48
CDC42BPB	139	180	144	99	126	105	37	33	48	34	43	49
CDC42BPG	6	2	2	3	1	0	0	1	1	0	4	1
CDC7	126	182	178	87	99	114	23	31	33	37	55	49
CDK1	336	438	374	252	251	314	58	80	65	56	168	160
CDK10	27	38	37	18	22	18	5	7	10	3	11	17
CDK11A	71	91	54	35	46	50	7	13	12	16	16	29
CDK12	265	393	301	203	216	247	65	56	66	93	87	88
CDK13	58	84	66	30	56	49	13	12	11	19	32	39
CDK14	0	2	1	0	1	0	1	1	2	0	1	3
CDK15	8	11	7	3	7	11	0	1	1	1	6	2

CDK16	80	163	101	63	60	64	16	27	19	27	46	57
CDK17	184	202	196	91	126	99	26	32	23	34	65	73
CDK18	5	5	2	1	4	1	4	4	1	2	2	1
CDK19	3	3	4	1	2	3	1	0	2	2	0	2
CDK2	6	11	7	3	4	7	3	2	2	1	4	4
CDK20	19	35	19	14	13	10	4	6	0	3	13	7
CDK3	6	3	3	2	0	1	0	3	2	2	1	2
CDK4	6	12	13	7	4	12	4	3	3	5	6	6
CDK5	192	381	211	124	183	179	32	44	22	34	121	144
CDK6	5	3	1	1	1	2	3	3	0	1	1	3
CDK7	632	759	608	319	420	421	107	93	74	115	268	351
CDK8	109	203	147	83	91	94	21	28	37	34	57	61
CDK9	8	15	7	10	6	7	1	2	6	1	4	14
CDKL1	4	2	1	4	4	3	0	5	3	1	4	6
CDKL2	4	2	5	3	2	5	3	6	4	1	2	6
CDKL3	5	11	7	4	2	9	3	1	1	2	3	4
CDKL4	3	4	9	7	5	2	2	1	3	1	4	3
CDKL5	24	34	21	27	30	32	6	3	8	15	19	30
CHEK1	410	565	474	321	293	308	83	107	102	107	117	142
CHEK2	53	78	50	31	46	46	19	24	14	14	24	30
CHUK	198	232	172	114	113	127	40	31	17	35	69	47
CIT	21	65	42	31	35	29	13	6	7	16	19	33
CLK1	291	387	266	151	216	224	44	50	40	51	164	125
CLK2	43	60	44	20	35	24	8	5	3	5	19	19
CLK3	68	102	98	52	49	58	24	14	17	14	28	34
CLK4	8	20	11	10	7	9	6	3	2	5	11	17
CLTC	54	92	50	31	48	52	11	14	14	14	28	31
COL4A3BP	52	100	66	39	70	57	8	12	14	10	29	57
CPNE3	3	1	0	0	4	2	2	1	1	0	0	3
CSF1R	67	116	43	41	23	38	14	7	14	12	32	54
CSK	87	123	67	51	40	55	13	22	15	12	23	28
CSNK1A1	114	139	91	58	65	69	13	16	13	16	33	37
CSNK1A1L	2	1	2	4	4	1	4	1	0	0	1	1

CSNK1D	272	559	298	185	200	216	73	74	66	68	139	167
CSNK1E	174	214	158	91	94	113	34	32	35	37	50	50
CSNK1G1	160	208	183	90	99	123	25	33	32	48	53	79
CSNK1G2	87	116	116	65	81	78	14	27	27	16	16	31
CSNK1G3	108	166	154	60	96	93	24	19	19	30	45	49
CSNK2A1	207	266	199	122	129	118	47	34	37	47	45	58
CSNK2A2	125	181	176	101	106	102	37	44	38	33	37	58
DAPK1	2	5	0	1	0	2	0	1	0	0	1	1
DAPK2	10	13	6	6	5	7	5	4	4	3	6	4
DAPK3	320	531	338	214	215	233	72	104	76	85	95	110
DCLK1	2	1	3	0	2	3	1	0	2	0	0	1
DCLK2	167	453	279	109	169	209	79	70	55	58	100	141
DCLK3	1	2	1	0	1	1	0	4	3	2	2	2
DDR1	86	86	59	58	40	52	20	15	22	31	16	49
DDR2	4	5	10	1	4	5	4	7	3	4	2	1
DMPK	5	5	2	3	5	1	4	1	2	0	3	1
DSTYK	6	9	9	2	10	6	0	1	1	0	2	3
DYRK1A	354	524	402	259	272	329	82	66	42	81	170	169
DYRK1B	0	4	2	0	1	3	0	2	1	1	2	1
DYRK2	34	52	63	45	32	45	11	5	16	21	19	32
DYRK3	28	43	45	27	29	35	3	5	6	18	3	23
DYRK4	2	9	2	1	1	0	2	3	2	1	4	1
EEF2K	93	141	111	73	70	66	27	15	14	30	48	34
EGFR	1	3	0	1	0	1	4	0	4	0	0	0
EIF2AK1	115	165	148	64	67	86	28	26	18	30	39	31
EIF2AK2	127	165	115	63	85	72	35	26	16	42	30	46
EIF2AK3	4	7	5	2	4	4	3	2	1	3	1	9
EIF2AK4	60	122	61	48	54	44	14	21	21	20	33	48
EPHA1	1	0	1	0	0	0	0	0	0	2	2	1
EPHA10	5	2	2	1	1	1	3	1	2	1	4	0
EPHA2	3	4	7	3	5	6	1	2	4	1	2	2
EPHA3	3	4	5	2	4	5	2	2	5	2	0	0
EPHA4	27	39	27	25	22	16	11	6	7	9	9	28

EPHA5	0	0	1	1	1	0	0	0	0	0	2	1
EPHA6	2	2	4	4	1	1	4	4	5	1	1	2
EPHA7	1	1	0	1	1	0	1	3	0	1	0	2
EPHA8	1	1	1	1	0	3	0	0	2	0	2	1
EPHB1	39	50	38	26	29	33	8	12	8	15	16	25
EPHB2	4	11	2	3	1	4	2	1	3	4	1	1
EPHB3	0	0	1	0	0	1	1	1	1	0	1	2
EPHB4	0	1	1	0	0	1	0	0	0	0	1	0
EPHB6	5	3	0	0	0	0	1	1	5	0	0	4
ERBB2	0	1	1	1	1	0	1	3	1	0	0	0
ERBB3	0	0	2	1	1	1	0	0	1	0	0	1
ERBB4	144	191	156	83	82	103	29	27	32	28	89	83
ERN1	130	205	221	108	100	104	29	22	44	41	60	43
ERN2	3	1	2	4	1	3	2	2	5	2	5	0
FASTK	6	8	4	1	4	4	3	4	1	0	6	6
FASTKD1	74	104	83	67	60	39	17	16	16	18	44	29
FASTKD2	27	43	20	19	23	25	7	5	4	7	14	18
FASTKD3	54	70	66	38	46	38	10	10	12	7	14	36
FASTKD5	6	5	9	4	6	5	1	5	4	2	3	10
FER	6	6	11	3	4	7	2	2	5	2	3	1
FES	5	4	4	0	2	3	3	3	3	2	2	5
FGFR1	120	148	125	68	81	75	17	29	23	26	37	49
FGFR2	52	53	50	20	34	28	15	13	14	12	16	27
FGFR3	8	4	1	1	1	2	4	3	3	7	3	9
FGFR4	1	2	4	2	1	0	0	1	1	0	1	0
FGFRL1	52	95	61	38	41	49	10	14	11	13	27	26
FGR	1	1	0	1	1	2	1	1	1	1	0	1
FLJ25006	3	3	4	4	0	4	1	1	4	3	1	0
FLT1	2	1	2	2	0	3	0	1	0	0	2	1
FLT3	1	5	3	2	4	1	6	2	5	1	1	2
FLT4	3	3	2	3	6	1	3	3	5	5	6	2
FRK	3	2	4	2	1	3	0	3	3	2	1	5
FYN	450	613	474	335	279	326	75	93	72	81	126	190

G6PD	106	164	112	85	82	72	28	21	21	34	45	70
GAK	40	109	52	61	44	35	18	16	26	19	19	25
GAPDH	216	314	198	127	127	192	45	59	62	54	94	137
GRK1	4	4	3	4	0	2	2	5	2	3	2	3
GRK4	0	4	1	0	1	1	0	0	2	2	1	0
GRK5	44	72	37	47	47	44	11	13	9	10	17	23
GRK6	18	32	18	12	14	8	7	10	5	5	14	15
GRK7	24	14	10	6	10	5	4	2	3	4	1	2
GSG2	41	58	35	27	35	35	7	13	10	12	17	24
GSK3A	269	414	317	180	206	179	61	49	76	77	94	118
GSK3B	76	158	137	51	78	68	25	23	22	28	35	49
GUCY2C	3	3	0	0	1	0	0	1	0	0	0	0
GUCY2D	16	11	6	9	6	5	2	7	4	3	3	5
GUCY2F	2	4	2	3	4	1	3	4	2	0	1	6
GUSB	6	22	10	6	5	2	2	3	3	1	1	4
HCK	0	1	2	0	0	3	1	0	4	0	2	0
HIPK1	38	61	54	42	59	40	9	14	14	18	38	37
HIPK2	44	68	37	25	47	51	13	12	4	8	23	30
HIPK3	19	22	36	28	28	27	6	4	8	7	24	25
HIPK4	80	99	55	29	13	40	21	14	5	10	18	24
HPRT1	426	467	410	195	218	273	66	72	38	65	95	130
HSPB8	1	0	1	0	0	1	0	2	0	0	0	0
HUNK	44	46	40	24	29	14	8	12	10	7	19	22
HUS1	23	36	38	17	17	24	4	6	7	4	15	12
ICK	19	23	19	6	11	10	6	6	7	5	11	6
IGF1R	245	351	251	140	176	175	71	49	62	62	100	91
IKBKB	30	34	27	15	22	17	2	2	9	8	12	7
IKBKE	1	15	6	0	10	6	0	3	3	1	1	2
ILK	3	12	5	2	6	4	0	2	3	2	4	1
INSR	18	6	11	10	4	5	2	2	2	0	2	2
INSRR	2	3	2	3	0	3	4	1	1	2	3	1
IRAK1	7	15	7	7	5	8	3	4	1	1	3	9
IRAK2	139	126	46	38	56	67	15	27	10	18	32	20

IRAK3	3	5	2	5	4	4	6	4	5	2	0	2
IRAK4	15	11	11	6	4	7	3	2	2	2	4	6
ITK	4	3	7	2	0	3	3	4	3	1	1	5
JAK1	83	123	84	63	55	62	14	18	23	12	22	49
JAK2	40	74	55	32	48	44	12	11	18	20	28	38
JAK3	6	2	1	4	2	4	4	4	3	3	9	1
KALRN	0	2	2	2	0	1	1	3	0	0	0	1
KDR	2	2	1	1	4	0	2	0	1	0	1	2
KIAA1804	16	6	8	8	4	3	1	0	0	5	3	4
KIT	152	172	149	96	117	87	13	23	19	32	32	79
KSR1	60	77	58	42	40	51	20	16	16	24	36	33
KSR2	12	22	6	16	16	13	4	5	3	3	11	5
LATS1	78	91	81	51	55	55	17	12	16	8	33	51
LATS2	69	116	91	88	75	67	10	16	14	25	30	56
LCK	1	1	2	5	5	1	2	3	3	0	1	1
LIMK1	117	182	121	90	111	98	30	36	21	28	63	57
LIMK2	73	131	70	55	67	53	17	20	16	13	38	39
LMTK2	36	67	50	27	36	32	15	15	18	30	30	33
LMTK3	2	4	5	2	5	1	2	2	4	1	5	6
LRRK1	8	17	10	9	10	8	5	7	10	8	11	8
LRRK2	0	0	0	0	0	2	0	0	2	2	1	1
LTK	9	9	4	5	13	10	1	3	4	3	5	3
LYN	2	2	0	1	0	0	0	1	2	0	3	1
MAK	44	70	54	32	37	33	5	5	12	13	17	34
MAP2K1	1459	1968	1383	883	976	984	295	282	231	325	460	449
MAP2K2	98	164	100	72	79	70	18	19	17	25	38	57
MAP2K3	112	142	121	63	109	67	14	40	32	22	51	49
MAP2K4	39	53	43	30	19	38	16	12	14	16	23	28
MAP2K5	20	26	8	7	5	10	4	7	5	3	11	7
MAP2K6	20	55	62	30	36	50	12	12	23	7	24	53
MAP2K7	201	286	227	118	124	113	43	56	40	39	49	63
MAP3K1	3	6	6	2	2	2	1	0	1	0	3	15
MAP3K10	31	54	41	28	29	21	9	13	5	9	14	11

MAP3K11	2	8	3	1	1	3	0	2	0	0	1	1
MAP3K12	14	20	16	1	6	10	4	6	1	4	1	3
MAP3K13	3	5	2	0	0	3	2	2	3	3	1	1
MAP3K14	11	17	13	15	14	10	3	4	5	4	3	6
MAP3K15	8	18	10	11	11	5	6	1	2	3	5	9
MAP3K2	3	2	1	1	1	1	0	0	0	0	0	1
MAP3K3	62	94	85	60	55	53	7	7	20	24	20	38
MAP3K4	82	90	89	49	56	55	9	15	26	11	16	24
MAP3K5	34	37	20	9	13	17	0	5	4	2	6	9
MAP3K6	0	0	0	0	0	0	0	0	0	1	0	0
MAP3K7	4	18	1	5	11	6	0	1	3	3	1	4
MAP3K8	1	1	3	3	2	2	2	1	1	0	2	0
MAP3K9	27	26	11	9	7	19	1	1	2	4	9	16
MAP4K1	1	1	3	1	1	3	2	1	1	1	1	1
MAP4K2	26	34	27	15	22	27	9	5	4	7	13	11
MAP4K3	112	155	122	65	111	90	19	29	22	27	40	56
MAP4K4	44	150	103	83	70	38	16	19	18	18	43	46
MAP4K5	47	69	63	35	24	32	13	17	4	12	26	22
MAPK1	49	82	55	29	49	43	10	6	20	11	37	45
MAPK10	10	16	11	3	7	7	2	5	3	2	7	5
MAPK11	0	2	2	2	0	0	0	2	1	0	0	2
MAPK12	79	94	88	55	60	52	16	24	14	14	29	34
MAPK13	13	11	10	6	5	12	7	3	4	4	7	6
MAPK14	20	38	43	24	24	24	9	4	7	13	16	16
MAPK15	2	0	0	1	0	2	0	1	1	1	1	1
MAPK3	21	21	11	10	11	16	13	5	3	2	9	8
MAPK4	100	95	96	45	70	66	20	22	14	7	31	34
MAPK6	197	388	255	136	111	182	60	53	20	35	77	99
MAPK7	54	125	58	61	54	50	18	13	5	13	50	37
MAPK8	192	244	200	102	141	136	34	44	37	46	66	59
MAPK9	65	58	56	39	43	32	12	4	12	13	11	15
MAPKAPK2	130	198	142	82	119	103	32	43	34	28	55	45
MAPKAPK3	0	1	0	0	1	1	1	0	3	0	0	0

MAPKAPK5	54	61	37	23	36	24	8	4	13	11	14	25
MARK1	9	0	3	4	2	2	1	1	0	1	4	7
MARK2	109	159	116	78	88	89	25	24	27	41	53	54
MARK3	58	71	54	27	48	44	7	7	14	15	19	34
MARK4	112	190	131	89	93	92	38	31	24	33	38	46
MAST1	0	0	0	0	1	0	1	0	0	0	0	0
MAST2	64	80	64	33	42	57	15	17	14	13	15	28
MAST3	25	12	14	9	8	11	7	4	4	5	5	2
MAST4	74	107	69	62	55	65	15	14	19	23	36	50
MASTL	125	148	86	56	87	68	20	16	19	34	55	82
MATK	24	53	34	25	13	19	6	6	5	7	5	10
MELK	719	813	634	458	451	503	149	136	89	192	239	271
MERTK	94	86	52	57	38	47	15	13	6	20	27	36
MET	0	3	1	0	0	3	0	2	4	1	4	1
MINK1	59	92	63	61	35	39	26	11	9	21	26	40
MKNK1	227	290	209	137	150	114	45	62	26	31	71	74
MKNK2	378	445	301	355	225	216	58	80	73	83	109	78
MLKL	9	36	8	7	10	12	7	4	3	9	6	6
MOK	107	49	128	51	59	44	5	10	27	16	30	44
MOS	381	583	388	197	474	262	46	69	71	105	171	220
MST1R	0	2	1	1	1	3	2	1	0	1	1	3
MST4	8	4	7	4	5	4	3	1	6	3	5	7
MTOR	92	142	95	50	75	58	22	19	21	15	33	26
MUSK	2	2	1	1	0	2	3	1	1	1	0	1
MYLK	29	61	32	27	22	23	8	9	8	7	9	12
MYLK2	2	2	0	1	0	2	2	1	0	0	2	0
MYLK3	0	1	2	1	1	1	1	0	0	2	2	0
MYLK4	3	3	2	1	1	3	1	0	0	1	3	1
MYO3A	1	1	1	1	2	1	0	0	1	1	1	4
MYO3B	60	84	53	37	30	32	12	15	14	8	35	23
NEK1	81	137	99	67	79	68	18	24	20	25	27	28
NEK10	1	2	1	2	1	2	2	2	4	0	2	1
NEK11	11	18	8	5	5	5	8	5	5	2	4	7

NEK2	273	316	309	171	171	168	43	68	54	51	91	70
NEK3	19	18	25	10	18	14	1	7	1	0	3	14
NEK4	62	89	63	47	48	51	14	7	11	16	15	25
NEK5	9	6	7	6	5	8	2	5	5	4	7	4
NEK6	5	9	2	5	2	3	0	0	1	0	5	0
NEK7	130	137	120	70	70	70	18	23	21	23	46	46
NEK8	6	8	16	7	4	7	3	3	4	2	9	6
NEK9	13	15	10	5	7	3	7	1	5	5	6	4
NIM1	1	0	2	0	1	2	0	1	0	1	2	0
NLK	61	69	56	41	47	33	14	11	13	11	23	22
NPR1	0	1	1	0	0	1	0	1	1	0	0	1
NPR2	1	2	1	1	0	3	1	1	0	0	1	1
NRBP1	143	251	163	97	99	95	22	29	18	34	70	104
NRBP2	7	5	10	5	4	8	3	2	3	3	1	3
NRK	0	2	0	1	4	2	0	0	0	3	0	1
NTRK1	1	3	1	0	0	0	2	1	1	1	2	1
NTRK2	1	1	2	0	0	0	0	1	0	1	1	0
NTRK3	3	2	0	1	0	1	1	0	1	1	1	3
NUAK1	1	1	0	0	0	0	0	1	1	0	0	0
NUAK2	9	20	18	8	6	5	2	3	3	5	6	16
OBSCN	0	0	3	3	1	3	2	0	0	1	2	2
OXSR1	30	43	27	18	17	25	8	4	5	7	15	14
PAK1	126	202	144	100	96	99	44	45	24	33	58	67
PAK2	53	74	50	41	50	45	5	7	10	15	17	26
PAK3	2	2	0	1	1	1	1	1	2	0	3	1
PAK4	62	110	79	43	44	63	14	14	14	12	27	37
PAK6	4	14	11	9	12	8	7	4	2	2	1	6
PAK7	13	21	10	7	13	18	4	2	3	2	4	6
PAN3	87	101	73	50	95	62	19	17	16	16	33	23
PASK	70	129	122	31	77	55	26	21	21	16	37	41
PBK	37	50	42	34	12	29	4	5	4	7	14	16
PDGFRA	6	2	4	5	5	6	9	3	5	3	4	3
PDGFRB	3	4	5	4	5	1	3	0	2	1	1	1

PDIK1L	49	54	55	30	46	35	6	9	3	10	16	37
PDK1	353	436	398	204	234	209	56	46	74	49	107	166
PDK2	89	134	137	34	53	69	17	29	15	16	45	47
PDK3	2	2	1	3	4	4	0	1	3	0	0	0
PDK4	2	0	0	0	1	2	0	1	1	2	1	0
PDPK1	70	85	48	23	34	46	16	22	9	12	22	44
PEAK1	117	136	109	103	72	92	25	23	32	32	48	55
PGK1	133	216	140	78	95	120	17	14	44	33	55	123
PHKG1	0	1	1	6	1	0	2	1	2	3	2	2
PHKG2	22	48	21	17	8	10	7	9	4	3	9	6
PIK3R4	10	15	17	10	12	11	2	4	1	3	12	18
PIM1	71	77	77	45	46	45	14	11	32	21	15	23
PIM2	6	20	9	12	8	7	2	3	3	8	7	22
PIM3	78	106	76	56	64	79	16	17	21	16	30	49
PINK1	46	52	29	22	18	25	5	5	6	9	11	23
PKDCC	6	10	4	1	2	4	1	1	2	1	1	2
PKMYT1	89	97	68	57	63	79	15	12	16	15	27	48
PKN1	65	103	80	39	40	58	10	18	11	14	22	38
PKN2	94	139	125	74	93	106	27	23	24	32	41	71
PKN3	6	8	8	3	5	0	3	1	2	1	3	3
PLK1	172	288	171	109	115	122	35	49	33	36	84	104
PLK2	6	8	1	1	4	8	5	2	4	1	2	2
PLK3	238	323	222	173	151	155	47	62	62	55	32	62
PLK4	28	32	14	12	19	16	4	7	3	3	13	17
PNCK	1	0	0	1	1	0	2	1	3	0	0	1
PRKAA1	39	42	33	33	36	26	3	4	11	12	15	39
PRKAA2	5	8	8	3	5	3	2	2	3	0	2	0
PRKACA	199	229	184	110	113	113	34	43	41	42	58	65
PRKACB	1	4	5	3	0	4	1	1	2	3	0	1
PRKACG	0	4	1	1	1	1	0	1	2	0	1	3
PRKCA	5	4	1	1	4	2	2	3	1	4	0	5
PRKCB	0	2	2	0	1	2	0	1	3	1	0	1
PRKCD	14	21	5	6	6	9	7	3	2	4	2	7

PRKCE	4	5	9	7	12	6	1	2	2	2	1	3
PRKCG	2	0	2	2	2	0	1	2	1	1	3	3
PRKCH	46	93	59	25	31	25	9	3	9	10	16	35
PRKCI	47	77	57	38	46	36	9	17	18	13	32	74
PRKCQ	53	83	57	34	26	35	11	21	10	13	13	29
PRKCZ	56	83	77	38	47	45	7	18	12	7	14	19
PRKD1	3	2	2	1	2	1	0	1	4	8	1	2
PRKD2	201	341	196	97	136	117	36	24	30	26	63	103
PRKD3	15	26	19	11	22	27	5	2	5	11	7	13
PRKDC	7	17	7	4	4	8	1	0	5	2	1	10
PRKG1	16	19	15	19	7	9	8	9	1	0	10	3
PRKG2	8	18	16	5	16	12	5	4	5	1	6	7
PRKX	0	2	1	0	0	0	3	0	2	1	1	0
PRKY	3	2	2	4	4	3	1	3	2	4	2	2
PRPF4B	12	31	22	6	20	17	4	4	5	1	5	13
PSKH1	21	25	14	10	6	15	7	11	11	9	5	9
PSKH2	3	2	0	3	1	1	1	0	0	2	2	4
PTK2	133	156	99	82	91	92	21	31	24	32	27	57
PTK2B	13	23	13	15	12	20	3	2	1	1	3	6
PTK6	2	3	0	1	0	2	0	1	1	2	2	1
PTK7	5	4	6	0	8	5	2	2	5	5	5	6
PXK	336	711	318	250	308	238	102	146	79	92	153	166
RAF1	3	2	0	0	1	1	1	0	1	1	2	1
RET	4	3	3	0	0	1	1	4	1	0	0	1
RIOK1	149	219	191	106	127	96	20	30	14	36	36	48
RIOK2	145	220	225	142	105	110	35	48	14	26	52	80
RIOK3	262	308	254	182	184	161	60	43	55	44	71	90
RIPK1	9	4	10	7	5	6	3	1	1	1	1	1
RIPK2	66	130	55	46	54	57	21	26	16	14	48	45
RIPK3	1	0	2	0	0	0	1	0	2	2	0	0
RIPK4	5	6	5	3	5	4	0	0	0	3	5	12
RNASEL	8	6	7	6	1	7	6	3	10	10	7	1
ROCK1	63	93	95	43	71	65	16	9	16	27	35	52

ROCK2	2	3	6	0	1	3	1	1	2	2	2	4
ROR1	6	6	4	4	6	4	3	4	3	0	3	3
ROR2	1	4	3	3	5	1	1	0	3	5	0	2
ROS1	45	146	139	82	49	108	25	28	23	45	48	60
RPS6KA1	32	69	41	28	36	24	17	23	11	5	20	21
RPS6KA2	5	8	13	5	5	1	3	3	3	2	3	3
RPS6KA3	3	5	0	2	1	2	0	1	0	0	2	2
RPS6KA4	2	0	0	1	0	0	1	0	1	1	0	0
RPS6KA5	54	80	67	34	40	50	4	16	25	13	16	25
RPS6KA6	20	32	15	11	20	16	3	2	3	11	12	17
RPS6KB1	19	36	21	14	22	13	1	3	4	11	10	18
RPS6KB2	7	12	11	4	10	8	0	4	1	1	2	7
RPS6KC1	249	355	225	187	169	166	66	51	76	56	69	77
RPS6KL1	19	51	30	16	24	13	10	7	5	8	5	9
RYK	5	7	5	5	5	3	2	0	3	3	1	3
SBK1	16	23	16	8	13	12	4	4	4	7	7	9
SBK2	0	4	2	0	1	2	0	1	0	0	0	0
SCYL1	83	134	67	51	49	60	18	20	12	12	33	52
SCYL2	97	124	86	48	44	46	14	13	7	18	26	33
SCYL3	58	68	55	30	52	44	7	9	14	15	21	23
SDHA	107	230	212	83	113	109	32	53	30	23	50	59
SGK1	23	31	24	15	29	24	2	1	3	7	12	109
SGK110	66	74	46	34	52	46	18	16	16	11	18	11
SGK196	311	386	393	265	299	291	58	70	68	99	123	115
SGK2	3	3	1	0	1	3	1	2	0	3	1	1
SGK223	3	3	2	1	2	1	1	1	0	3	0	3
SGK3	11	25	30	18	20	10	5	4	6	0	11	13
SGK494	6	6	2	5	4	3	1	5	5	5	6	5
SIK1	25	31	15	25	23	17	5	5	8	5	10	16
SIK2	29	45	29	22	29	21	6	15	5	8	7	14
SIK3	18	32	16	17	18	23	5	3	7	4	6	19
SLK	46	46	61	22	30	32	7	15	3	7	9	19
SMG1	171	229	166	103	149	140	36	23	33	60	77	109

SNRK	28	30	17	18	12	24	2	7	3	2	13	5
SPEG	3	3	3	1	0	1	0	0	0	1	1	1
SRC	8	5	10	8	7	6	5	6	5	5	3	5
SRMS	1	1	1	0	0	0	1	0	2	2	1	4
SRPK1	420	550	461	248	332	282	79	89	81	85	119	135
SRPK2	97	111	118	69	63	76	13	15	32	46	45	66
SRPK3	0	0	0	1	0	1	1	2	0	0	0	1
STK10	20	20	10	10	8	11	2	4	2	0	7	13
STK11	88	149	77	65	76	65	19	16	19	20	32	43
STK16	11	15	14	4	13	10	3	2	5	4	4	7
STK17A	3	4	4	7	6	3	0	0	3	1	6	19
STK17B	48	69	61	27	32	40	7	7	11	12	12	28
STK19	34	26	26	12	20	17	6	7	2	7	12	10
STK24	1402	1704	1200	584	1072	883	279	324	252	219	460	287
STK25	108	218	104	64	100	95	19	38	20	19	44	62
STK3	11	6	12	7	4	9	2	1	0	3	3	3
STK31	54	64	42	29	38	48	8	7	5	13	28	19
STK32A	1	3	4	2	2	3	0	0	1	0	1	3
STK32B	149	196	138	100	87	116	24	26	32	34	47	45
STK32C	13	20	19	11	20	7	6	3	2	5	5	10
STK33	73	84	82	49	54	40	12	13	13	5	31	38
STK35	261	359	260	205	185	229	31	50	53	74	96	97
STK36	23	36	20	20	17	19	9	7	2	13	10	12
STK38	114	178	128	96	111	104	30	27	25	43	35	42
STK38L	38	37	27	25	29	33	7	2	8	15	9	23
STK39	84	171	110	64	84	76	25	30	14	27	53	61
STK4	34	35	33	10	19	29	7	7	9	9	13	8
STK40	65	114	61	41	55	46	22	17	8	11	47	63
STRADA	47	117	64	43	54	49	12	14	8	15	40	64
STRADB	43	125	65	38	47	56	15	10	13	5	20	27
STYK1	4	36	15	10	5	8	8	2	5	2	4	4
SYK	3	2	1	3	4	1	1	1	2	0	1	1
TAF1	40	54	60	33	35	35	11	7	10	9	11	11

TAF1L	42	74	48	45	28	38	13	9	11	14	26	18
TAOK1	203	337	217	128	130	164	40	43	51	53	96	84
TAOK2	171	229	133	95	123	100	27	35	27	26	31	64
TAOK3	203	297	284	181	173	147	25	53	64	57	87	93
TBCK	44	53	44	37	18	23	11	11	6	9	18	16
TBK1	58	69	80	35	53	65	9	14	13	8	28	44
TBRG4	32	45	33	27	26	24	4	15	7	1	9	17
TEC	0	1	0	0	0	1	1	0	2	1	2	0
TEK	38	69	33	30	23	42	10	11	7	11	28	25
TESK1	134	195	178	109	119	116	26	31	24	32	41	68
TESK2	39	43	40	17	28	20	5	4	10	5	22	29
TEX14	33	42	28	17	13	17	6	7	7	10	15	21
TGFBR1	64	64	64	48	79	69	7	6	13	5	19	67
TGFBR2	7	2	4	3	2	7	2	1	6	2	6	3
TIE1	0	0	1	1	1	1	1	0	2	0	0	0
TLK1	21	25	24	26	19	17	6	1	4	7	10	20
TLK2	73	100	88	62	59	72	18	18	22	27	22	48
TNIK	1	2	4	3	4	3	1	3	5	2	1	3
TNK1	1	3	1	0	1	3	0	0	2	1	2	0
TNK2	67	92	65	59	59	56	9	4	18	20	18	23
TNNI3K	0	1	0	0	0	3	1	0	0	0	1	1
TP53RK	4	3	2	1	0	3	0	1	0	0	1	5
TRIB1	2	0	2	0	0	2	0	0	0	0	0	1
TRIB2	11	8	2	6	6	4	2	6	2	0	2	3
TRIB3	2	2	1	2	2	1	0	0	1	3	1	1
TRIM24	20	22	17	12	14	17	5	7	13	1	10	22
TRIM28	108	148	87	115	102	91	12	13	32	27	74	128
TRIM33	245	367	219	152	226	175	49	45	57	51	82	170
TRIO	435	652	471	324	404	312	107	76	112	119	169	221
TRPM6	2	0	0	1	0	2	0	0	1	0	0	1
TRPM7	94	114	91	54	58	67	21	13	17	28	28	43
TRRAP	69	66	76	75	56	52	16	12	14	18	21	41
TSSK1B	0	4	4	1	4	0	4	1	5	3	1	3

TSSK2	0	1	1	2	1	2	2	2	3	2	0	0
TSSK3	2	1	2	0	0	1	2	0	2	2	1	3
TSSK4	7	5	5	6	4	3	6	4	5	2	0	4
TSSK6	0	2	0	2	1	1	2	0	2	3	0	3
TTBK1	5	2	5	4	0	1	4	1	1	1	1	5
TTBK2	62	68	50	35	44	45	7	6	14	12	14	14
TTK	579	729	556	367	362	379	127	116	88	120	189	219
TTN	1	3	0	1	1	2	0	1	0	0	1	2
TUBB	6	4	1	2	1	3	2	1	2	1	1	1
TXK	0	0	3	2	0	1	0	0	0	0	0	1
TYK2	4	9	5	5	7	7	2	3	0	2	0	1
TYRO3	2	1	1	1	1	3	0	1	0	0	3	2
UHMK1	139	190	188	117	137	135	31	45	53	45	65	69
ULK1	112	150	111	78	67	79	15	20	29	22	30	34
ULK2	90	179	115	82	105	83	20	36	23	39	64	67
ULK3	81	122	88	49	69	54	15	41	33	13	23	30
ULK4	155	187	317	210	108	125	36	34	23	35	58	59
VRK1	202	247	198	100	141	149	37	45	32	36	66	62
VRK2	58	91	69	45	52	44	5	12	10	12	13	25
VRK3	93	94	100	64	70	56	12	17	29	19	30	43
WEE1	5	3	4	1	2	2	1	0	1	1	2	8
WEE2	7323	10170	8929	5811	5630	5639	1758	1639	1620	1911	2645	2662
WNK1	78	138	76	49	58	59	23	12	11	26	40	71
WNK2	11	28	14	19	2	13	2	9	9	11	2	6
WNK3	16	12	18	9	19	11	4	5	1	8	5	7
WNK4	4	2	2	3	6	1	2	3	2	2	0	2
YES1	674	777	655	422	513	481	124	127	157	182	260	248
YSK4	2	1	1	1	0	0	0	0	0	2	0	0
ZAK	1	7	4	3	2	8	4	2	1	3	4	2
ZAP70	1	3	1	0	0	0	1	1	0	0	0	1
