# Supplementary material for: Digital multiplexed mRNA analysis of functionally important genes in single human oocytes and correlation of changes in transcript levels with oocyte protein expression
Source: Fertil Steril. 2014 Mar;101(3):857–64. doi: 10.1016/j.fertnstert.2013.11.125 (PMC3969224; doi:10.1016/j.fertnstert.2013.11.125)
Supplement: Supplemental Table 2 [file mmc2.pdf]

Positive Control Normalised Counts

Oocyte Nos.	"One-thirds"			Single-oocytes			Single-oocytes			"Empty"		
Target												
POS_A(128)	37487	37514	37140	37306	37359	37271	37373	37407	37279	37318	37238	37335
POS_B(32)	6467	6534	6796	6693	6556	6745	6686	6711	6760	6694	6764	6716
POS_C(8)	2010	1986	2085	2001	2048	1998	1957	1895	2024	1967	1986	1942
POS_D(2)	598	541	554	569	596	555	554	583	536	584	577	590
POS_E(0.5)	144	145	145	144	153	149	146	125	133	154	152	141
POS_F(0.125)	43	29	31	35	36	31	34	29	18	33	32	26
NEG_A(0)	0	0	0	0	0	1	1	0	0	0	0	0
NEG_B(0)	5	3	2	2	1	0	0	2	1	0	1	1
NEG_C(0)	0	2	2	2	1	5	1	4	3	3	2	0
NEG_D(0)	26	25	28	26	29	26	28	34	25	31	27	35
NEG_E(0)	1	0	1	2	2	0	1	1	0	1	1	0
NEG_F(0)	1	1	3	2	0	2	2	3	1	0	1	0
NEG_G(0)	0	0	0	0	0	0	0	0	0	0	0	2
NEG_H(0)	0	2	0	0	1	1	2	1	2	0	0	0
AAK1	14	13	12	10	10	9	8	5	11	0	0	0
AATK	4	0	1	2	2	2	4	1	1	0	4	0
ABL1	37	35	19	17	22	28	13	26	16	0	1	0
ABL2	26	19	18	18	15	17	10	19	11	0	0	0
ACVR1	24	16	14	7	6	9	14	12	16	0	0	1
ACVR1B	50	53	61	44	32	52	37	42	42	0	0	0
ACVR1C	0	0	1	1	0	1	0	1	2	0	0	0
ACVR2A	22	19	23	14	11	17	12	16	11	0	0	0
ACVR2B	37	29	36	32	42	33	33	40	32	0	1	1
ACVRL1	0	1	0	2	1	2	0	1	2	1	1	2
ADCK1	1	1	1	1	1	1	3	2	0	2	1	0

ADCK2	24	17	31	14	16	19	15	23	17	0	0	0
ADCK3	4	1	6	1	0	4	1	5	1	1	0	1
ADCK4	1	1	0	0	2	1	1	1	3	0	0	1
ADCK5	1	0	1	0	1	0	3	1	0	0	0	2
ADRBK1	50	30	44	29	41	47	39	43	34	2	1	0
ADRBK2	8	5	8	11	9	11	8	6	3	0	0	0
AKT1	56	49	58	52	64	59	45	69	44	0	1	1
AKT2	92	79	86	52	71	73	48	73	62	2	2	1
AKT3	97	79	93	95	87	91	79	90	81	2	1	1
ALK	4	1	3	1	1	0	0	0	2	2	1	0
ALPK1	1	0	0	0	2	1	0	2	0	0	0	0
ALPK2	0	2	1	1	1	0	0	0	0	0	0	0
ALPK3	0	1	1	0	1	1	0	1	0	0	0	0
AMHR2	2	0	0	1	1	2	0	1	2	0	1	0
ANKK1	2	1	0	0	0	1	1	0	0	2	1	2
ARAF	11	9	13	8	13	12	6	7	7	2	1	1
ATM	0	0	0	2	3	3	0	0	2	2	0	1
ATR	6	4	18	4	8	9	2	6	3	0	0	0
AURKA	477	418	456	328	378	391	254	357	240	1	2	0
AURKB	37	33	53	28	18	30	9	42	19	0	0	0
AURKC	85	57	77	44	42	43	23	36	32	1	2	1
AXL	4	0	0	0	0	3	4	0	2	2	0	1
BCKDK	8	7	7	5	4	4	3	4	6	2	2	2
BCR	36	37	49	34	34	37	31	34	22	1	0	1
BLK	12	10	7	6	1	3	7	16	6	0	1	0
BMP2K	29	21	29	15	19	24	14	27	15	0	0	1
BMPR1A	96	86	94	63	64	66	37	67	50	0	0	0
BMPR1B	10	10	5	9	7	16	9	15	9	1	0	0
BMPR2	25	17	30	13	17	22	20	10	8	0	1	1
BMX	0	2	0	1	0	0	0	0	2	2	0	0
BRAF	12	12	8	4	7	6	4	12	11	6	1	2
BRD2	69	56	37	45	50	57	25	46	46	0	0	0
BRD3	17	12	18	19	13	21	12	19	13	0	1	0

BRD4	98	78	114	74	94	91	72	96	80	3	1	5
BRDT	14	12	26	12	10	12	7	17	1	0	1	1
BRSK1	2	1	0	1	0	1	0	4	2	0	0	2
BRSK2	1	1	0	1	0	0	0	1	1	1	1	0
BTK	0	0	0	1	0	0	1	2	1	1	0	0
BUB1	44	44	36	32	30	29	13	52	18	0	2	1
BUB1B	187	140	204	156	140	130	86	153	48	2	1	0
C21orf7	67	69	78	28	42	46	5	48	8	2	1	0
C9orf96	1	0	0	0	0	0	2	1	0	0	1	1
CAMK1	2	0	1	0	3	0	0	2	2	1	0	1
CAMK1D	11	11	11	11	10	9	9	6	13	2	1	0
CAMK1G	0	2	0	2	1	1	1	0	0	1	0	1
CAMK2A	5	3	4	2	4	1	2	12	3	2	2	0
CAMK2B	0	0	0	0	0	1	0	1	0	0	1	1
CAMK2D	1	0	1	3	4	2	0	0	4	0	0	2
CAMK2G	73	47	39	34	34	46	25	37	25	4	3	0
CAMK4	5	9	10	7	6	6	4	8	3	3	1	0
CAMKK1	1	1	2	3	0	1	5	4	1	2	0	1
CAMKK2	6	5	6	3	1	3	2	2	3	2	1	1
CAMKV	0	0	0	1	1	1	1	3	0	0	1	0
CASK	1	0	0	1	0	1	0	0	1	1	1	0
CCL2	1	1	5	3	0	0	4	0	1	3	3	0
CDC42BPA	28	19	25	15	10	19	9	14	10	2	2	0
CDC42BPB	36	37	40	33	37	28	27	37	29	5	4	2
CDC42BPG	0	0	2	0	0	1	1	0	0	0	1	0
CDC7	34	27	19	25	25	13	12	28	22	0	0	0
CDK1	129	97	127	92	64	83	46	111	23	0	0	2
CDK10	12	9	6	12	8	10	6	7	4	1	0	4
CDK11A	18	13	15	14	15	20	5	8	8	0	1	0
CDK12	97	89	77	63	72	61	42	68	45	4	1	0
CDK13	24	14	19	10	25	17	13	11	9	0	0	0
CDK14	2	4	0	1	1	0	1	2	0	0	0	1
CDK15	1	1	4	1	1	2	2	6	3	1	0	0

CDK16	41	31	24	11	20	19	16	21	20	2	0	0
CDK17	56	47	40	31	36	24	19	22	16	0	1	0
CDK18	1	1	0	1	2	0	0	0	1	1	1	1
CDK19	0	0	2	2	3	0	1	1	2	2	2	2
CDK2	1	0	4	2	1	0	2	2	0	0	0	0
CDK20	7	3	6	3	4	2	3	6	1	0	1	0
CDK3	1	1	1	0	0	0	0	1	0	0	0	0
CDK4	2	1	5	2	3	3	2	2	2	1	2	0
CDK5	58	54	43	45	38	31	31	37	20	0	0	0
CDK6	0	0	1	1	2	0	1	0	1	2	2	1
CDK7	230	173	209	138	95	102	36	99	54	0	0	0
CDK8	32	26	36	21	29	28	12	21	13	1	0	0
CDK9	1	1	2	3	0	2	2	3	1	0	1	0
CDKL1	5	1	0	0	2	0	5	1	0	2	2	0
CDKL2	0	0	1	1	2	2	1	0	1	1	2	1
CDKL3	2	4	3	1	2	6	0	4	1	2	0	2
CDKL4	4	1	1	2	2	2	0	1	2	1	1	0
CDKL5	14	7	10	5	5	3	11	6	5	2	2	0
CHEK1	103	101	112	86	100	96	53	87	61	0	0	0
CHEK2	25	18	27	18	6	13	8	26	6	4	2	7
CHUK	61	37	52	53	36	35	17	44	21	1	1	0
CIT	12	13	16	9	10	9	4	6	3	1	0	1
CLK1	97	107	124	67	60	60	34	51	46	3	0	2
CLK2	6	7	6	5	4	3	1	6	5	0	0	0
CLK3	25	17	24	11	24	25	11	17	14	0	0	1
CLK4	2	5	6	3	7	3	4	2	0	0	1	0
CLTC	20	18	18	9	12	14	7	13	4	0	0	0
COL4A3BP	32	18	24	12	17	23	18	15	13	2	2	1
CPNE3	4	0	2	2	0	0	1	1	1	3	0	0
CSF1R	16	16	10	8	5	7	9	20	7	1	2	1
CSK	24	9	12	15	10	7	8	10	7	1	0	0
CSNK1A1	30	18	27	21	29	22	13	16	10	0	0	1
CSNK1A1L	1	3	1	0	0	0	0	2	2	1	1	0

CSNK1D	121	93	105	75	79	85	51	77	53	3	3	1
CSNK1E	44	34	42	35	33	44	18	45	27	2	3	0
CSNK1G1	47	39	58	37	34	38	20	37	17	0	0	1
CSNK1G2	22	23	16	25	29	25	20	13	14	2	1	0
CSNK1G3	36	29	30	19	16	20	14	29	15	0	0	0
CSNK2A1	54	42	45	33	34	44	35	46	27	0	0	0
CSNK2A2	44	23	32	30	33	28	35	46	34	1	0	1
DAPK1	0	0	0	0	1	1	1	1	0	1	0	0
DAPK2	2	3	2	1	4	1	2	1	1	2	0	0
DAPK3	102	80	91	51	72	69	58	66	62	0	0	1
DCLK1	1	0	2	1	0	1	1	0	0	0	0	0
DCLK2	99	76	68	54	70	65	24	58	28	0	1	0
DCLK3	1	1	2	2	1	0	1	0	1	1	1	0
DDR1	28	12	25	10	14	14	11	18	11	5	1	2
DDR2	4	0	2	4	1	0	3	1	1	2	3	2
DMPK	2	0	2	0	1	2	0	0	1	3	2	0
DSTYK	2	2	2	2	1	2	2	3	0	0	1	0
DYRK1A	132	123	109	60	71	72	45	77	40	0	0	0
DYRK1B	1	0	3	0	0	0	0	0	0	1	0	0
DYRK2	23	15	11	8	14	9	15	14	12	1	1	0
DYRK3	16	8	8	5	12	7	5	12	2	1	0	0
DYRK4	0	1	1	2	1	1	1	0	2	0	2	2
EEF2K	32	14	19	27	15	17	5	12	14	0	0	1
EGFR	0	2	1	0	2	1	0	0	1	1	0	1
EIF2AK1	43	37	31	10	24	19	19	20	12	1	1	5
EIF2AK2	49	29	39	17	23	33	17	17	18	1	0	1
EIF2AK3	4	2	1	1	0	2	0	2	1	1	0	2
EIF2AK4	22	14	24	8	15	16	15	24	14	1	1	1
EPHA1	0	1	0	0	0	0	0	0	0	0	0	0
EPHA10	0	0	1	1	2	0	0	0	0	0	1	0
EPHA2	0	4	3	0	0	1	0	0	1	0	0	5
EPHA3	2	0	0	2	1	2	2	1	1	1	0	0
EPHA4	6	5	7	11	8	8	7	6	11	1	2	0

EPHA5	0	0	1	0	0	0	1	0	0	0	0	0
EPHA6	0	1	3	3	2	3	2	0	0	2	3	2
EPHA7	1	0	3	0	1	1	1	0	0	0	0	0
EPHA8	1	0	0	0	0	2	0	0	0	0	1	1
EPHB1	10	12	8	6	10	10	6	15	9	0	0	0
EPHB2	1	0	1	0	1	2	3	1	2	1	1	0
EPHB3	0	0	0	0	0	0	1	1	0	1	0	0
EPHB4	0	1	0	0	1	0	0	0	0	1	0	0
EPHB6	1	0	1	0	0	0	1	1	0	1	0	1
ERBB2	0	0	0	0	0	0	0	0	0	1	0	0
ERBB3	0	1	0	0	0	0	1	1	0	1	0	0
ERBB4	28	40	55	30	35	39	21	35	25	0	0	1
ERN1	66	44	70	31	30	39	43	22	42	0	2	1
ERN2	1	1	2	1	1	2	1	2	0	3	1	0
FASTK	0	2	1	1	1	1	1	2	0	0	0	1
FASTKD1	17	16	15	21	9	20	16	24	5	0	0	0
FASTKD2	5	8	8	6	2	5	6	3	4	1	1	1
FASTKD3	23	19	15	11	11	8	4	15	4	0	0	0
FASTKD5	1	0	1	2	0	1	1	0	1	2	1	1
FER	2	5	1	2	5	0	0	0	1	0	1	0
FES	1	0	0	3	2	0	1	0	1	2	0	0
FGFR1	17	26	32	26	14	40	22	30	23	0	1	0
FGFR2	22	22	15	10	12	10	13	15	20	13	11	13
FGFR3	1	1	1	0	2	1	4	1	4	3	4	5
FGFR4	0	2	0	0	0	3	0	0	1	0	0	0
FGFRL1	12	20	17	11	9	12	14	18	10	0	0	0
FGR	1	0	0	0	1	1	1	1	0	1	0	0
FLJ25006	0	1	2	0	4	0	0	1	1	1	0	1
FLT1	0	0	0	1	0	1	0	1	1	1	1	2
FLT3	1	1	0	1	0	2	1	1	1	1	4	1
FLT4	2	1	1	2	5	2	1	3	5	0	0	4
FRK	0	0	2	1	2	1	1	0	1	1	2	0
FYN	151	113	127	100	72	81	57	97	52	0	0	2

G6PD	31	34	38	22	24	29	17	40	17	0	0	1
GAK	17	18	17	21	20	35	20	15	18	0	1	1
GAPDH	50	46	68	27	49	50	26	45	31	0	3	4
GRK1	0	1	2	0	1	2	0	0	2	1	1	1
GRK4	0	0	1	0	0	0	1	0	0	0	1	0
GRK5	14	17	14	9	9	6	8	12	4	0	0	1
GRK6	6	3	6	1	5	4	2	2	3	1	2	4
GRK7	1	4	3	3	4	3	2	1	3	0	0	1
GSG2	11	13	21	7	14	13	11	15	10	2	2	0
GSK3A	102	83	82	52	75	73	53	69	52	1	1	1
GSK3B	37	29	27	18	29	20	15	18	19	2	1	0
GUCY2C	0	1	0	0	0	0	0	1	1	0	0	0
GUCY2D	4	3	5	1	2	7	6	2	3	0	2	0
GUCY2F	2	2	1	2	0	3	4	2	3	3	3	4
GUSB	1	3	4	5	3	1	1	9	2	0	1	1
HCK	0	1	0	1	2	1	2	0	1	0	1	0
HIPK1	24	17	18	24	14	21	11	12	8	0	2	0
HIPK2	18	5	12	10	12	9	7	14	4	0	2	0
HIPK3	13	13	15	9	8	11	7	5	7	2	3	0
HIPK4	19	19	7	9	9	15	6	8	5	0	0	2
HPRT1	85	66	71	58	60	80	30	79	22	0	0	0
HSPB8	0	0	0	0	0	0	2	0	0	2	1	1
HUNK	11	4	10	12	15	11	8	12	3	1	1	0
HUS1	14	5	4	9	7	9	5	8	4	0	0	1
ICK	6	9	5	1	2	1	2	5	2	0	0	0
IGF1R	56	52	55	54	51	63	35	64	40	0	0	0
IKBKB	12	5	6	4	7	12	1	3	4	1	1	0
IKBKE	4	1	3	3	5	0	0	1	0	0	1	0
ILK	2	3	1	2	0	2	1	1	0	0	0	0
INSR	6	7	4	3	4	5	0	1	3	4	2	0
INSRR	0	0	4	1	0	0	1	0	1	0	1	1
IRAK1	4	0	1	3	1	3	3	0	2	0	1	0
IRAK2	18	22	30	11	8	7	10	15	19	1	0	1

IRAK3	1	5	1	1	0	1	3	1	3	3	1	1
IRAK4	1	2	2	2	4	3	2	3	1	0	0	1
ITK	0	0	0	0	0	2	2	1	1	0	0	1
JAK1	16	14	19	19	27	20	21	17	14	0	0	0
JAK2	10	18	18	11	11	11	10	13	15	0	3	0
JAK3	1	0	2	0	1	0	2	4	0	0	1	2
KALRN	1	0	1	1	1	1	2	0	1	0	3	2
KDR	1	0	0	0	0	0	1	1	0	0	1	2
KIAA1804	2	0	1	3	1	4	3	0	1	1	2	0
KIT	36	38	58	26	26	27	14	42	28	1	0	1
KSR1	24	10	15	9	9	7	10	17	17	1	0	1
KSR2	5	2	2	4	2	1	2	3	3	0	1	0
LATS1	23	20	31	24	18	20	9	18	14	1	1	1
LATS2	17	18	19	13	21	30	13	14	13	0	0	2
LCK	0	1	1	2	1	1	3	0	1	1	2	1
LIMK1	43	37	45	23	27	29	12	28	11	0	0	0
LIMK2	17	21	15	9	12	17	13	15	10	0	0	0
LMTK2	34	18	26	3	12	9	8	11	4	1	1	1
LMTK3	1	1	0	2	1	0	3	0	2	3	2	0
LRRK1	2	7	6	6	8	5	2	6	10	1	3	2
LRRK2	2	1	1	0	1	1	2	0	1	0	0	1
LTK	1	0	1	1	4	3	1	2	8	2	0	2
LYN	0	1	0	1	1	0	1	0	1	0	0	0
MAK	19	12	10	8	12	9	11	2	14	1	1	0
MAP2K1	341	335	369	243	291	268	188	275	214	1	1	0
MAP2K2	38	34	45	24	24	26	22	32	15	0	1	0
MAP2K3	43	31	34	28	25	39	26	52	27	0	2	2
MAP2K4	13	12	6	11	10	17	7	11	5	2	4	2
MAP2K5	4	1	6	2	4	2	3	0	2	0	2	2
MAP2K6	14	7	6	9	8	14	23	14	26	3	1	2
MAP2K7	31	47	36	27	43	41	31	45	35	0	0	0
MAP3K1	1	1	3	0	0	1	1	1	0	0	0	0
MAP3K10	6	8	6	5	10	6	7	13	5	1	0	1

MAP3K11	4	1	0	1	1	1	2	0	1	0	0	0
MAP3K12	6	3	5	1	4	0	3	2	5	0	1	0
MAP3K13	1	3	1	1	1	1	0	3	1	1	0	2
MAP3K14	2	2	5	4	5	0	8	2	2	0	2	1
MAP3K15	1	7	6	3	4	6	3	7	1	0	1	0
MAP3K2	0	1	0	0	0	0	0	1	0	0	0	0
MAP3K3	28	12	18	11	21	17	11	18	15	1	0	0
MAP3K4	24	28	32	12	16	24	27	17	18	1	1	0
MAP3K5	2	3	6	5	8	5	0	6	0	0	1	0
MAP3K6	0	0	0	0	0	1	0	0	0	0	0	0
MAP3K7	4	4	6	5	6	3	1	2	3	0	0	2
MAP3K8	0	0	2	1	0	0	0	0	1	2	0	0
MAP3K9	5	6	6	3	5	5	4	6	2	0	0	0
MAP4K1	0	1	3	1	1	0	0	0	2	1	2	0
MAP4K2	7	4	6	8	4	3	5	4	7	0	0	0
MAP4K3	28	34	41	23	21	26	12	23	8	0	0	2
MAP4K4	32	19	36	12	26	24	3	13	2	0	0	0
MAP4K5	14	20	19	6	11	9	4	19	13	0	0	0
MAPK1	30	18	18	8	21	11	13	18	10	0	0	0
MAPK10	1	4	4	2	1	5	2	0	0	0	0	0
MAPK11	0	1	0	0	0	0	2	0	1	2	1	0
MAPK12	18	18	18	17	24	13	14	12	11	0	1	1
MAPK13	2	2	5	3	2	0	0	1	3	2	3	1
MAPK14	18	13	10	2	5	7	8	9	8	0	1	0
MAPK15	0	1	2	0	0	2	0	0	0	0	0	1
MAPK3	4	3	7	3	4	3	7	6	5	2	0	1
MAPK4	35	31	23	20	21	18	17	29	22	1	2	1
MAPK6	32	15	45	21	16	20	22	45	19	0	0	0
MAPK7	19	26	16	9	16	10	8	17	6	0	0	2
MAPK8	49	44	56	40	30	34	27	44	25	1	1	0
MAPK9	17	19	16	15	12	17	11	14	8	0	1	0
MAPKAPK2	43	39	35	38	36	39	31	38	21	0	1	0
MAPKAPK3	0	0	0	2	2	0	0	1	0	0	0	0

MAPKAPK5	14	12	5	5	12	10	8	10	9	0	1	0
MARK1	4	1	1	0	3	0	2	1	0	1	0	0
MARK2	24	29	36	31	29	27	20	33	21	1	2	0
MARK3	17	18	19	6	11	10	4	12	11	0	0	0
MARK4	34	36	35	20	33	32	19	46	21	1	1	2
MAST1	0	0	0	2	0	0	0	0	0	0	0	0
MAST2	14	11	12	6	10	6	9	15	13	1	0	0
MAST3	11	2	6	2	4	5	0	4	2	1	0	0
MAST4	37	19	21	11	18	17	8	15	8	0	0	0
MASTL	43	34	34	29	27	17	14	33	15	0	1	0
MATK	5	10	3	5	5	8	3	4	3	0	0	0
MELK	217	169	198	182	152	142	57	187	46	10	9	11
MERTK	20	15	15	19	11	13	13	22	6	0	0	1
MET	0	1	0	2	0	2	1	0	0	2	1	0
MINK1	19	23	11	8	13	22	15	15	15	1	0	0
MKNK1	42	41	46	60	61	41	27	40	24	0	0	0
MKNK2	110	99	75	70	71	68	63	92	64	1	2	1
MLKL	5	2	5	0	3	1	2	6	2	1	0	4
MOK	16	17	31	24	29	25	20	22	21	2	1	1
MOS	161	110	137	81	93	82	36	63	28	1	1	4
MST1R	0	0	0	0	1	2	1	3	0	0	2	1
MST4	1	1	1	0	4	1	1	0	3	3	2	1
MTOR	17	13	23	18	20	30	12	21	8	0	0	0
MUSK	0	0	2	1	1	0	0	1	0	1	0	1
MYLK	16	12	11	3	8	7	8	14	13	2	1	1
MYLK2	1	3	0	0	0	0	2	0	1	0	0	0
MYLK3	0	2	0	0	0	0	1	1	0	0	0	1
MYLK4	0	0	1	0	0	0	0	0	2	0	1	0
MYO3A	0	0	2	0	0	2	3	0	1	0	0	1
MYO3B	19	11	16	7	14	17	7	19	6	0	0	0
NEK1	38	28	22	26	26	32	11	24	17	0	4	0
NEK10	0	3	2	3	3	3	0	1	0	2	2	2
NEK11	1	3	0	0	2	0	4	0	4	1	3	1

NEK2	74	79	81	18	23	46	35	77	30	0	0	0
NEK3	6	4	6	2	4	3	6	4	5	2	1	1
NEK4	13	16	22	15	11	17	6	12	18	1	0	1
NEK5	1	2	0	2	1	5	2	2	2	1	1	4
NEK6	1	2	1	1	2	0	0	0	1	0	0	0
NEK7	43	39	35	23	25	32	19	17	28	2	6	0
NEK8	1	3	3	0	5	1	2	0	2	1	1	0
NEK9	4	6	1	3	2	2	5	2	2	1	1	0
NIM1	0	0	1	0	0	0	0	1	0	0	0	1
NLK	20	22	18	18	11	20	14	8	13	0	2	2
NPR1	0	1	1	2	0	1	0	0	0	0	0	0
NPR2	0	1	0	1	1	2	1	0	1	1	0	1
NRBP1	31	23	32	22	29	28	13	30	15	0	0	0
NRBP2	0	2	0	3	3	3	2	2	1	0	0	0
NRK	0	1	0	1	0	0	0	0	4	0	0	0
NTRK1	1	1	0	0	0	0	1	0	0	0	0	1
NTRK2	0	1	0	0	2	0	1	1	0	1	0	0
NTRK3	0	1	1	0	0	0	0	1	2	0	1	2
NUAK1	0	0	0	0	1	1	0	0	0	0	0	1
NUAK2	2	2	3	4	3	1	1	2	4	3	0	1
OBSCN	0	2	1	1	0	1	0	0	0	0	0	1
OXSR1	8	12	9	5	9	6	2	7	3	0	0	1
PAK1	41	29	49	26	39	31	22	49	26	0	1	1
PAK2	13	17	15	14	12	9	8	13	6	1	2	0
PAK3	0	0	1	1	0	1	0	0	1	0	1	1
PAK4	28	16	25	15	12	21	8	16	6	0	0	0
PAK6	4	7	4	2	6	4	2	2	2	1	1	1
PAK7	1	1	2	2	3	4	8	1	3	0	3	2
PAN3	20	19	24	20	27	11	15	21	17	0	2	0
PASK	29	26	21	15	18	17	8	15	11	0	0	0
PBK	7	6	17	7	10	6	0	6	6	0	0	0
PDGFRA	1	0	2	0	2	1	0	2	0	2	3	4
PDGFRB	1	0	2	4	2	3	4	1	3	2	2	0

PDIK1L	12	8	9	7	7	9	6	8	5	0	0	1
PDK1	101	76	90	65	75	60	42	60	45	2	2	0
PDK2	42	34	40	29	28	32	18	16	20	1	2	4
PDK3	1	3	0	0	3	0	1	3	0	0	1	0
PDK4	1	0	0	0	2	2	0	0	0	1	0	0
PDPK1	23	18	22	8	10	9	4	21	2	0	3	1
PEAK1	40	26	36	25	22	35	23	25	27	0	2	0
PGK1	48	60	55	30	42	38	25	43	35	0	0	1
PHKG1	5	1	3	0	3	2	2	1	2	2	0	0
PHKG2	6	4	4	5	6	6	5	8	7	0	1	0
PIK3R4	7	4	8	2	4	5	2	2	1	1	1	0
PIM1	26	23	27	8	11	9	16	15	15	1	2	0
PIM2	6	1	6	3	2	1	3	5	3	2	2	1
PIM3	26	16	23	14	16	21	9	19	7	0	0	0
PINK1	4	9	8	3	6	5	3	7	7	5	1	0
PKDCC	1	1	2	2	0	1	1	0	1	0	0	0
PKMYT1	18	35	23	24	21	16	12	8	11	0	1	0
PKN1	23	20	16	9	12	12	13	13	11	0	0	0
PKN2	41	22	36	32	25	39	22	19	18	0	0	2
PKN3	2	2	6	2	1	3	1	1	3	2	1	0
PLK1	72	47	48	27	31	35	22	38	19	0	0	0
PLK2	0	1	2	1	0	0	2	4	1	1	0	0
PLK3	84	53	73	52	42	44	39	50	35	0	1	2
PLK4	4	9	7	2	6	4	8	10	5	1	1	2
PNCK	0	2	1	0	1	1	1	1	0	1	2	0
PRKAA1	12	10	12	5	8	6	6	6	7	3	0	0
PRKAA2	0	1	2	2	1	1	1	0	0	1	0	0
PRKACA	58	41	59	34	47	43	23	51	38	2	1	1
PRKACB	1	0	1	1	1	1	2	1	2	2	1	0
PRKACG	0	1	0	0	0	1	1	1	1	1	0	0
PRKCA	0	3	0	1	0	1	2	1	3	1	2	0
PRKCB	1	2	2	1	2	1	2	3	0	0	1	1
PRKCD	5	6	3	2	1	4	2	3	1	0	2	0

PRKCE	0	0	1	1	1	3	1	2	0	0	1	0
PRKCG	1	0	0	3	2	0	1	0	0	2	3	1
PRKCH	17	14	10	3	10	6	5	12	4	0	1	0
PRKCI	19	21	13	12	7	19	10	18	16	2	4	2
PRKCQ	14	14	12	8	10	12	10	11	10	0	0	1
PRKCZ	13	7	12	9	11	8	15	11	4	0	0	0
PRKD1	1	0	1	0	0	0	1	1	1	1	2	0
PRKD2	43	39	64	28	46	37	17	44	18	0	0	0
PRKD3	8	5	4	2	1	3	4	6	4	0	0	0
PRKDC	5	7	3	2	1	1	1	1	4	0	0	1
PRKG1	2	2	6	3	2	6	4	3	0	0	0	0
PRKG2	6	3	1	4	4	2	3	1	2	1	0	0
PRKX	1	3	1	0	1	0	0	0	0	1	1	0
PRKY	2	3	1	3	2	0	0	1	1	1	3	5
PRPF4B	2	3	6	3	5	6	2	5	7	0	1	0
PSKH1	1	7	7	4	3	6	2	4	8	4	3	1
PSKH2	0	3	1	0	3	1	3	0	0	0	2	0
PTK2	19	35	27	19	31	32	17	26	19	0	0	1
PTK2B	5	2	5	1	1	5	6	1	1	0	0	0
PTK6	1	1	0	0	0	0	0	0	0	0	0	0
PTK7	4	0	1	3	4	1	4	0	2	3	0	0
PXK	143	141	144	42	57	46	37	90	29	1	1	1
RAF1	0	0	2	0	0	1	1	0	0	0	0	0
RET	1	2	0	0	0	0	1	0	1	2	0	4
RIOK1	28	22	31	22	23	19	17	30	5	0	1	0
RIOK2	62	51	58	57	50	50	24	51	24	1	1	1
RIOK3	61	73	65	60	65	69	48	46	45	1	2	1
RIPK1	2	0	1	2	1	2	2	1	3	1	1	0
RIPK2	20	19	21	12	15	25	12	25	9	0	0	0
RIPK3	0	0	1	0	0	0	0	0	1	0	0	0
RIPK4	0	3	3	2	1	2	3	0	2	1	1	2
RNASEL	2	2	6	4	0	6	2	1	5	6	4	5
ROCK1	32	25	20	17	17	24	13	17	12	0	0	0

ROCK2	2	1	2	1	2	1	0	0	1	0	0	1
ROR1	0	3	1	0	4	2	2	3	1	3	2	0
ROR2	0	0	2	2	1	3	2	1	2	1	2	0
ROS1	48	32	44	12	30	32	11	35	32	1	0	0
RPS6KA1	10	10	10	10	10	15	11	13	6	2	1	0
RPS6KA2	1	1	2	4	0	4	0	2	1	0	0	0
RPS6KA3	1	1	0	0	0	0	0	0	0	0	0	0
RPS6KA4	0	0	1	1	1	1	1	1	0	1	1	0
RPS6KA5	23	21	10	9	10	13	4	11	9	0	0	1
RPS6KA6	8	6	2	6	4	4	1	3	5	0	2	1
RPS6KB1	8	9	6	3	2	6	1	4	4	2	0	0
RPS6KB2	0	5	1	1	3	2	1	1	4	0	1	0
RPS6KC1	73	65	71	56	89	97	36	74	49	2	0	0
RPS6KL1	5	6	6	2	10	4	3	5	5	1	0	1
RYK	4	0	2	2	0	0	2	0	3	0	0	0
SBK1	2	2	1	2	3	4	2	1	3	0	2	0
SBK2	0	0	2	0	1	0	0	2	1	1	1	0
SCYL1	26	14	26	17	17	20	19	18	6	1	2	2
SCYL2	28	17	28	8	12	13	6	19	13	0	0	0
SCYL3	11	19	15	5	13	6	8	14	7	0	2	0
SDHA	34	22	28	22	25	24	20	30	13	1	0	1
SGK1	7	6	11	5	3	2	2	1	0	0	1	1
SGK110	18	9	15	13	10	12	7	18	15	4	2	0
SGK196	90	77	74	78	84	83	58	71	60	1	2	1
SGK2	1	0	2	0	0	2	3	2	2	2	0	1
SGK223	0	0	2	1	2	0	3	1	0	2	0	1
SGK3	5	4	5	8	3	2	6	7	1	1	1	4
SGK494	2	4	1	2	2	1	4	2	1	1	1	2
SIK1	7	4	11	5	2	3	5	5	1	0	1	0
SIK2	12	7	6	3	4	1	5	8	7	0	0	1
SIK3	1	5	10	1	9	4	3	7	2	1	0	0
SLK	13	11	14	8	10	11	8	9	4	1	0	1
SMG1	58	44	57	39	39	38	27	49	31	0	0	0

SNRK	5	7	6	6	2	3	3	4	1	1	0	0
SPEG	0	0	1	1	2	0	0	0	0	0	0	0
SRC	2	2	4	1	2	3	7	4	3	2	1	2
SRMS	1	0	0	1	1	2	2	0	1	0	1	4
SRPK1	86	86	105	75	94	94	59	106	67	1	1	0
SRPK2	32	27	30	18	24	17	21	18	20	1	1	0
SRPK3	1	0	2	1	0	0	0	0	0	0	0	0
STK10	7	6	3	5	6	3	1	7	7	1	0	1
STK11	30	24	28	20	24	20	7	16	10	0	1	1
STK16	4	2	3	2	0	3	2	2	5	0	0	1
STK17A	1	2	2	1	5	1	1	4	1	1	0	1
STK17B	11	11	14	14	11	5	8	13	7	1	2	0
STK19	10	8	6	5	7	5	2	6	6	3	0	0
STK24	355	273	279	198	241	165	161	145	169	2	0	1
STK25	25	22	31	13	15	25	12	25	12	0	0	0
STK3	1	4	3	1	0	3	0	2	0	2	1	0
STK31	14	10	14	13	13	9	7	16	5	0	0	1
STK32A	0	0	1	1	1	0	0	0	0	0	1	1
STK32B	29	18	29	36	34	31	39	20	23	0	4	4
STK32C	1	2	6	1	3	2	1	2	3	0	0	0
STK33	30	20	29	14	9	9	10	20	7	1	1	2
STK35	66	77	82	47	70	58	49	63	49	0	0	1
STK36	18	15	8	2	2	0	1	10	1	0	1	0
STK38	37	41	50	21	30	40	22	34	24	0	0	0
STK38L	5	7	7	8	10	5	11	5	6	6	2	2
STK39	24	27	28	30	23	19	8	34	20	0	0	0
STK4	7	7	5	8	8	6	5	15	6	1	0	0
STK40	30	17	26	10	13	14	4	14	3	0	0	0
STRADA	26	17	16	11	16	13	7	15	3	0	0	0
STRADB	13	10	18	6	5	15	6	16	7	0	0	0
STYK1	1	0	2	1	5	5	1	5	2	1	0	0
SYK	1	2	0	0	3	1	1	0	1	0	0	0
TAF1	8	5	6	4	6	9	6	9	6	0	0	0

TAF1L	18	17	11	11	8	15	5	12	8	3	0	0
TAOK1	73	68	77	54	61	68	23	66	29	0	0	1
TAOK2	32	31	45	33	32	34	18	30	28	1	0	1
TAOK3	64	60	58	59	68	64	48	76	40	0	0	0
TBCK	10	10	6	7	13	4	8	10	5	1	1	0
TBK1	20	18	26	19	13	17	10	13	5	4	0	0
TBRG4	17	13	10	6	9	6	4	8	5	0	0	0
TEC	1	0	0	0	0	0	0	0	0	1	1	2
TEK	19	10	8	5	5	4	4	10	5	1	0	1
TESK1	61	41	46	39	40	34	27	45	43	0	0	0
TESK2	12	11	12	5	9	10	4	6	9	1	0	1
TEX14	5	7	6	3	3	3	7	10	6	2	0	0
TGFBR1	25	10	18	5	17	9	7	23	4	0	0	0
TGFBR2	1	1	3	0	3	3	3	2	4	4	1	2
TIE1	0	0	0	0	0	0	1	0	0	0	1	0
TLK1	12	7	10	3	7	6	6	11	1	0	2	0
TLK2	32	20	21	13	19	10	19	19	16	2	1	1
TNIK	0	2	1	0	1	2	1	1	0	0	0	0
TNK1	0	0	0	0	0	0	0	0	0	0	0	0
TNK2	16	12	9	13	20	16	17	20	19	0	0	0
TNNI3K	0	0	3	0	0	0	0	0	1	1	1	0
TP53RK	0	1	2	2	1	1	0	1	1	1	2	0
TRIB1	1	0	0	0	1	0	0	0	0	0	0	0
TRIB2	0	3	1	0	3	1	0	1	0	3	4	2
TRIB3	0	1	1	0	0	0	0	0	0	1	1	0
TRIM24	7	6	5	1	4	9	3	5	11	2	2	1
TRIM28	30	33	33	15	26	30	15	46	20	4	1	0
TRIM33	74	55	75	43	44	46	32	51	33	0	1	2
TRIO	140	120	163	117	108	96	80	122	96	1	0	0
TRPM6	0	0	0	1	1	0	0	0	1	1	2	0
TRPM7	23	20	19	24	19	21	15	20	18	0	2	1
TRRAP	18	14	21	10	10	10	28	16	21	0	1	0
TSSK1B	1	0	0	1	0	3	0	0	0	1	0	2

TSSK2	1	2	1	1	1	3	1	0	0	0	0	1
TSSK3	0	0	0	0	0	0	0	0	0	0	1	1
TSSK4	4	3	3	0	2	0	3	1	1	3	2	0
TSSK6	0	1	2	1	1	1	0	1	3	0	1	1
TTBK1	1	0	0	0	0	0	1	1	1	1	2	0
TTBK2	12	15	11	9	9	15	9	11	10	0	0	0
TTK	168	141	154	119	133	132	74	134	53	0	0	4
TTN	0	1	0	0	1	0	1	1	0	0	0	0
TUBB	1	2	1	1	1	1	1	3	2	1	1	1
TXK	0	0	0	0	1	0	0	0	0	0	0	0
TYK2	0	1	1	0	1	0	1	2	1	0	0	0
TYRO3	0	0	1	0	0	1	1	0	1	0	0	0
UHMK1	46	29	57	52	46	42	34	27	28	1	0	0
ULK1	20	18	30	22	21	19	20	35	15	0	0	0
ULK2	52	36	36	32	36	28	25	48	28	4	1	1
ULK3	25	26	28	9	17	19	20	19	22	1	1	0
ULK4	60	54	39	43	32	21	26	36	26	0	0	0
VRK1	58	44	56	38	40	32	20	49	24	0	0	0
VRK2	16	20	19	10	17	11	3	15	5	0	0	0
VRK3	20	21	22	23	17	17	17	26	9	0	0	1
WEE1	2	0	2	1	0	0	0	1	0	0	1	1
WEE2	2318	2017	2230	1975	1963	2124	1474	1821	1372	2	0	0
WNK1	25	33	34	17	22	17	10	20	15	0	3	1
WNK2	13	4	5	4	5	5	3	3	5	1	2	1
WNK3	8	1	4	4	2	7	6	3	3	1	1	1
WNK4	0	0	2	0	1	3	4	4	0	1	1	0
YES1	173	171	186	136	131	115	77	130	102	1	0	1
YSK4	0	1	1	0	0	0	1	0	0	1	1	0
ZAK	1	3	3	1	3	2	0	2	1	0	1	0
ZAP70	1	0	1	1	0	2	0	0	1	1	1	0
