# Supplementary figures and images for: Digital multiplexed mRNA analysis of functionally important genes in single human oocytes and correlation of changes in transcript levels with oocyte protein expression
Source: Fertil Steril. 2014 Mar;101(3):857–64. doi: 10.1016/j.fertnstert.2013.11.125 (PMC3969224; doi:10.1016/j.fertnstert.2013.11.125)

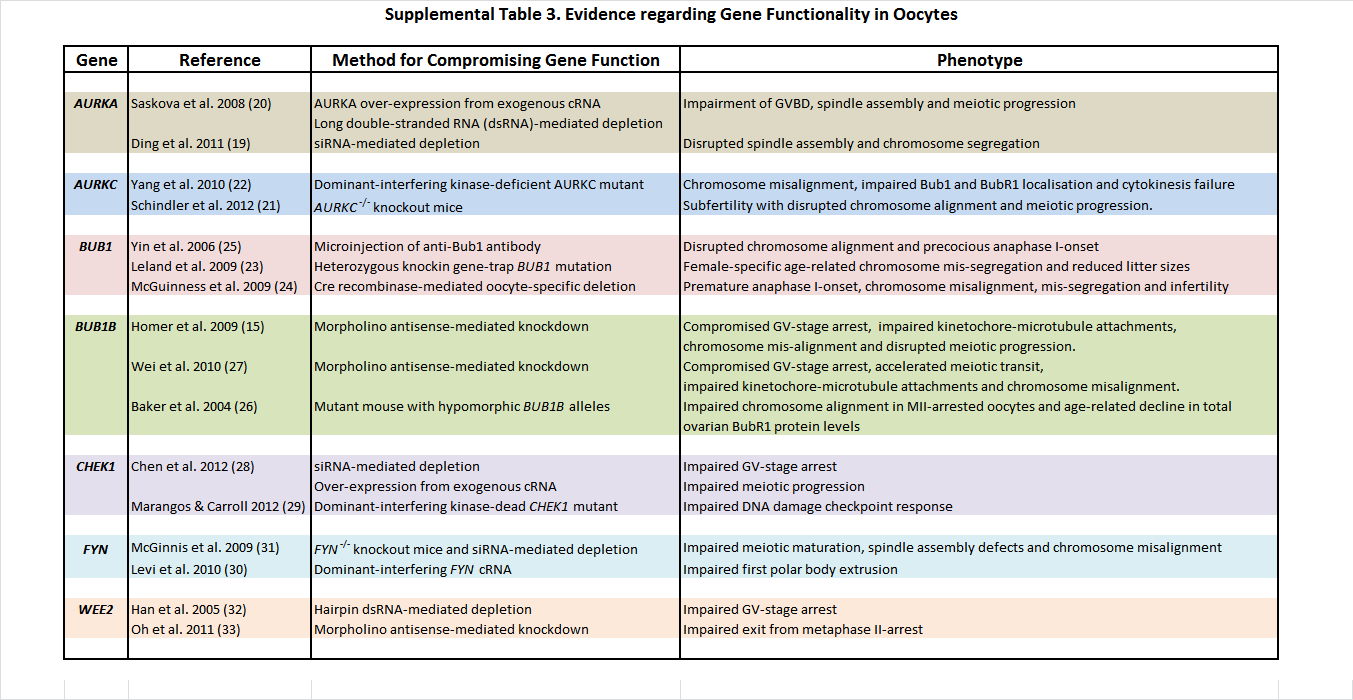

Supplement: Supplemental Table 3 [file mmc3.doc]
